# Optimal type and dose of hypoxic training for improving maximal aerobic capacity in athletes: a systematic review and Bayesian model-based network meta-analysis

**DOI:** 10.3389/fphys.2023.1223037

**Published:** 2023-09-05

**Authors:** Xinmiao Feng, Linlin Zhao, Yonghui Chen, Zihao Wang, Hongyuan Lu, Chuangang Wang

**Affiliations:** ^1^ Sports Coaching College, Beijing Sports University, Beijing, China; ^2^ Beijing Sport University, Beijing, China; ^3^ Capital Institute of Physical Education and Sports, Beijing, Beijing, China

**Keywords:** altitude/hypoxic training, maximal aerobic capacity, athletes, dose-response correlation, network meta-analysis

## Abstract

**Objective:** This study aimed to compare and rank the effect of hypoxic practices on maximum oxygen consumption (VO_2_max) in athletes and determine the hypoxic dose-response correlation using network meta-analysis.

**Methods:** The Web of Science, PubMed, EMBASE, and EBSCO databases were systematically search for randomized controlled trials on the effect of hypoxc interventions on the VO_2_max of athletes published from inception until 21 February 2023. Studies that used live-high train-high (LHTH), live-high train-low (LHTL), live-high, train-high/low (HHL), intermittent hypoxic training (IHT), and intermittent hypoxic exposure (IHE) interventions were primarily included. LHTL was further defined according to the type of hypoxic environment (natural and simulated) and the altitude of the training site (low altitude and sea level). A meta-analysis was conducted to determine the standardized mean difference between the effects of various hypoxic interventions on VO_2_max and dose-response correlation. Furthermore, the hypoxic dosage of the different interventions were coordinated using the “kilometer hour” model.

**Results:** From 2,072 originally identified titles, 59 studies were finally included in this study. After data pooling, LHTL, LHTH, and IHT outperformed normoxic training in improving the VO_2_max of athletes. According to the P-scores, LHTL combined with low altitude training was the most effective intervention for improving VO_2_max (natural: 0.92 and simulated: 0.86) and was better than LHTL combined with sea level training (0.56). A reasonable hypoxic dose range for LHTH (470–1,130 kmh) and HL (500–1,415 kmh) was reported with an inverted U-shaped curve relationship.

**Conclusion:** Different types of hypoxic training compared with normoxic training serve as significant approaches for improving aerobic capacity in athletes. Regardless of the type of hypoxic training and the residential condition, LHTL with low altitude training was the most effective intervention. The characteristics of the dose-effect correlation of LHTH and LHTL may be associated with the negative effects of chronic hypoxia.

## Introduction

Altitude training (AT) has been employed by well-trained athletes for almost 60 years since the 1968 Mexico Olympics (Buskirk et al., 1967; Wilber, 2007; Girard et al., 2023). Currently, this method has led to the development of several matured variants following the conventional “live-high train-high” approach (HH), i.e., living and training at low or moderate altitudes throughout the day (Buskirk et al., 1967). In contrast to the HH approach, the training site for “live-high train-low” (HL) is set at sea level or low altitudes (Levine and Stray-Gundersen, 1997; Bonetti and Hopkins, 2009). Besides, given the type of hypoxia in residential environments, HL can be simulated and natural (Coppel et al., 2015; Girard et al., 2023). However, considering the time involved in HH and HL training, IHE has induced wide attention over the past few years for its shorter but more intense hypoxia protocols during rest (Millet et al., 2010; Wilber, 2007). Training under short-term hypoxia is defined as an LLTH practice, which includes varying forms (e.g., intermittent hypoxic training [IHT]) (McLean et al., 2014). Live-high and train-high/low (HHL) has been reported as a novel hypoxic combination that is implemented during the application of HL, where athletes train under normoxic and hypoxic conditions (Millet et al., 2010).

Although AT has been extensively applied and is trusted by coaches and athletes (Daniels and Oldridge, 1970; Solli et al., 2017), its effectiveness on aerobic performance remains unclear (Millet and Brocherie, 2020; Siebenmann and Dempsey, 2020). In general, the ambiguity originates from two aspects: 1) Potential negative effects of chronic hypoxia exposure (various types of acute altitude sickness) (Imray et al., 2010; Schommer et al., 2012), which outweighs potential benefits and 2) unreasonable hypoxic doses (Wilber et al., 2007). Given the high cost of AT, its effectiveness should be determined, and a reasonable dosage range should be set. Although existing meta-analyses and dose-effect research have partially addressed the above-mentioned issues, most may contain flaws and controversies.

1. The above-described studies were inconclusive since there rarely was direct evidence on the comparison of different types of AT and since they failed to report a clear hierarchy (Bonetti and Hopkins, 2009; Hamlin et al., 2018; Lobigs et al., 2018)
2. Existing research has suggested that exposure to altitudes above 299 m negatively affects endurance performance; The risk of negative effects arising from hypoxic exposure becomes higher as the elevation rises, particularly above 1,000 m (Hamlin et al., 2015). However, most existing research has overlooked the actual hypoxic effect at low altitudes, and none of them has considered the potential differences in total hypoxic dose and benefits according to different training altitudes, including low altitude and sea level training sites of HL (Bonetti and Hopkins, 2009; Lancaster and Smart, 2012; Gore et al., 2013).
3. The continuity and severity of exposure are critical determinants of effective hypoxic dosage, and the deficiency in the study by Gore et al. lies in exploring the hypoxic dose-effect relationship only at a fixed altitude (Gore et al., 2013). The “kilometer hour” model proposed by Laura et al. can address the above limitation (Garvican-Lewis et al., 2016).

Accordingly, to fill the gap in comparing the effect of a wide variety of AT variants on athletes’ aerobic performance and explore reasonable hypoxic dose ranges, a network meta-analysis was conducted in this study. We aimed to compare and rank different hypoxic types for the improvement of VO_2_max in athletes. Subsequently, a novel technique (Bayesian model-based dose-response network meta-analysis) was adopted to examine the dose-response correlation between hypoxic doses and VO_2_max in athletes.

## Methods

This review followed the Preferred Reporting Items for Systematic Reviews and Meta-Analyses for Network Meta-Analyses (PRISMA-NMA) guidelines (Shamseer et al., 2015). Moreover, this study is registered with PROSPERO (registration number: CRD42023401488).

### Search strategy

The Web of Science, PubMed, EMBASE, and EBSCO databases were systematically searched for eligible studies published from database inception until 21 February 2023. The search terms [(hypoxia OR altitude OR hypoxic) AND (maximal oxygen consumption OR maximal oxygen uptake OR peak oxygen uptake OR maximal aerobic capacity)] were used for the search in “all fields,” whereas the terms (patients OR obese) were excluded (using NOT). Additional trials were retrieved from the cited references of relevant published meta-analyses and systematic reviews.

### Inclusion and exclusion criteria

The inclusion criteria were as follows: 1) randomized and non-randomized controlled trials with at least one group completing hypoxic training (this was to increase generalizability and allow for a wider range of evidence); 2) studies that included athletes without a history of smoking, injuries, and hypoxic experience during the past 6 months; 3) studies that used only AT without additional intervention (e.g., blood flow restriction or heat environment); and 4) studies with outcomes including maximal aerobic capacity direct testing/not estimated performed at or near sea level.

The exclusion criteria were as follows: studies that were not available in English; that were conference abstracts, experience, comments, animal, or case experiments; with populations that were non-athletes; with irrelevant outcomes (e.g., disease, oxidative disorders, and sleep quality); and that assessed acute effects of altitude/hypoxic conditions.

### Data extraction and quality evaluation

Studies that met the inclusion criteria were independently selected by two researchers. Subsequently, the main text and Supplementary Material S1 of the selected articles were independently reviewed to extract relevant information from the included trials. Moreover, the extracted studies were evaluated and rated by three reviewers based on the Cochrane Risk of Bias tool. Any differences between the reviewers in the above process were resolved through consensus and arbitration with other investigators in the review team.

The extracted information comprised publication information (e.g., first author, publication year, and title), study design (experimental type), study participants (e.g., sample size, projects, and sex), intervention measures [e.g., altitude mode, hypoxic dose (altitude, m and duration, h)], and outcome measures (test results of maximum oxygen uptake in a plain environment before and after the intervention). The means and standard deviations of the outcome measures data were extracted. When the required data could not be extracted, the authors attempted to contact the authors of the included studies at least three times within 4 weeks to request for the information.

### Intervention coding and hypoxic dose model

The hypoxic dosage of the different hypoxic types was coordinated using the “kilometer hour” model (Garvican-Lewis et al., 2016), defined as kmh = (m/1,000)× h, where “m” represents the altitude of the exposure environment and “h” represents the total exposure duration. In this study, the altitude/hypoxic training methods used in the included studies were categorized into the following three levels: 1) encoding based on the methodological design of altitude/hypoxic training, including HH, HL, IHT, IHE, and HHL; 2) subsequently, based on the first level encoding, we further refined the categorization of HL according to the type of hypoxia in the residential environment and the altitude of the training locations as follows: including living in a real altitude (hypobaric hypoxia) and training at a low altitude of 1,000–1500 m (HL_NAT/TLA), training in a simulated altitude (normobaric hypoxia) and training at a low altitude of 1,000–1500 m (HL_SIM/TLA), and living in a simulated altitude (normobaric hypoxia) and training at an altitude near sea level at <600 m (HL_SIM/TSL). In addition, we expanded the altitude range of the HH and included some control groups (which are not usually explicitly defined as HH in research) that underwent experiments at low altitudes into the HH category. We used a random-effects network meta-analysis within a Bayesian framework to rank and evaluate the intervention effects of various measures at this level; and 3) finally, the third level of encoding was also based on the first level, where the intervention measures were encoded at the intersection of specific types and dosages of AT, and the random-effects Bayesian Model-Based Network MetaAnalysis (MBNMA) was applied at this level. Furthermore, to facilitate the meta-analysis connectivity of the third level, the AT methods were condensed into several groups based on the approximate values of the hypoxic dosage (detailed data presented in Supplementary Annex S1).

### Statistical analysis

#### Network meta-analysis

The netmeta package in R software (version 3.6.3) was adopted to perform network meta-analysis, combining direct and indirect comparisons in the frequency model (Rücker, 2012; Shim et al., 2019). The standardized mean difference (SMD) and 95% confidence interval (CI) were employed as effect size indicators. A random-effect network meta-analysis model was adopted to assess the combined magnitude of the effect sizes of the included studies. In the network evidence plot of the network meta-analysis, the size of the dots represents the sample size, and the lines between the dots represent direct comparisons between the two types of exercise interventions. The increase in the thickness of the line indicate more direct comparisons between the two interventions; otherwise, the line is thinner. If there was no line between the two types of exercise interventions, an indirect comparison was performed using network meta-analysis. In addition, all paired comparisons of the combined SMD and 95% CI are presented in the league table, and the effect of the respective exercise interventions on the maximal oxygen uptake of the participants relative to the control group is presented in a forest plot. P-scores were adopted to rank the exercise types based on the improvement of athletes’ maximal oxygen uptake. The P-scores ranged from 0 to 1, with a higher score suggesting a greater improvement in aerobic capacity (Rücker and Schwarzer, 2015). Tau-squared (τ2) tests and *p*-values were employed to qualitatively analyze heterogeneity among the studies. The higher the τ2 value and the smaller the *p*-value, the greater the likelihood of heterogeneity; in a contrast situation, the smaller the heterogeneity. Moreover, heterogeneity among the results of the respective studies was quantitatively analyzed using I^2^. Its values ranged from 0% to 100%. When I^2^ was less than 25%, it indicated low heterogeneity; 25%–50% indicated moderate heterogeneity; and >75% indicated high heterogeneity. In brief, when I^2^ was >50%, there was significant heterogeneity. Global and local methods were employed to test the inconsistency of study results. Global inconsistency was evaluated through the design-by-treatment test (Higgins et al., 2012). Furthermore, local inconsistency tests were performed using the node-splitting method in the R netmeta package (Dias et al., 2010). Next, the statistical analysis of funnel plots was conducted using Egger’s method. A *p*-value less than 0.05 indicated publication bias. The sensitivity of this study was evaluated by repeating the respective network meta-analysis after the exclusion of studies with high risk of publication bias.

### Model-based network meta-analysis

Network meta-analysis based on random-effect Bayesian-based MBNMA (Mawdsley et al., 2016) was conducted to summarize the dose-response correlation between hypoxic dose and VO_2_max. No evidence of violation of the critical assumptions of network meta-analysis (Ter Veer et al., 2019) and consistency and transitivity of the data was reported (Wheeler et al., 2010; White et al., 2012) (Supplementary File S2). All effect sizes were reported as Hedges’ SMD (Ozawa and Onodera, 2017), and the credibility of the estimates of this study was evaluated using 95% credible intervals (CrI) (Etzioni and Kadane, 1995). First, the observed effects of different altitude/hypoxic training types and hypoxic doses on VO_2_max were plotted. A series of recommended nonlinear functions such as Emax, random or non-random restricted cubic spline, non-parametric, and exponential models (Pedder et al., 2019) were adopted to model the data based on the observed shape. Next, we derived and compared different fitting indices (Evans, 2019) (i.e., deviance information criterion [DIC], standard deviation between studies, number of parameters in the model, and residual values) and the corresponding bias plots (Evans, 2019). The random-effects model with restricted cubic splines produced the optimal fit in all cases (Supplementary File S2) and was, therefore, used to evaluate the nonlinear dose-response correlation (Harrell, 2017; Hamza et al., 2021). Departure from linearity was evaluated using Wald’s test (Harrell, 2017; Hamza et al., 2021). The hypoxic dose at which the restricted cubic spline had the greatest significant impact on VO_2_max was evaluated based on the Beta coefficient. Capitalizing on novel meta-analytic techniques (i.e., model-based dose-response network meta-analysis under a Bayesian framework) and evidence stemming from existing controlled trials, the current report examined the dose-response correlation between hypoxic dose and the VO_2_max of athletes.

## Results

### Characteristics of included studies

**FIGURE 1:**
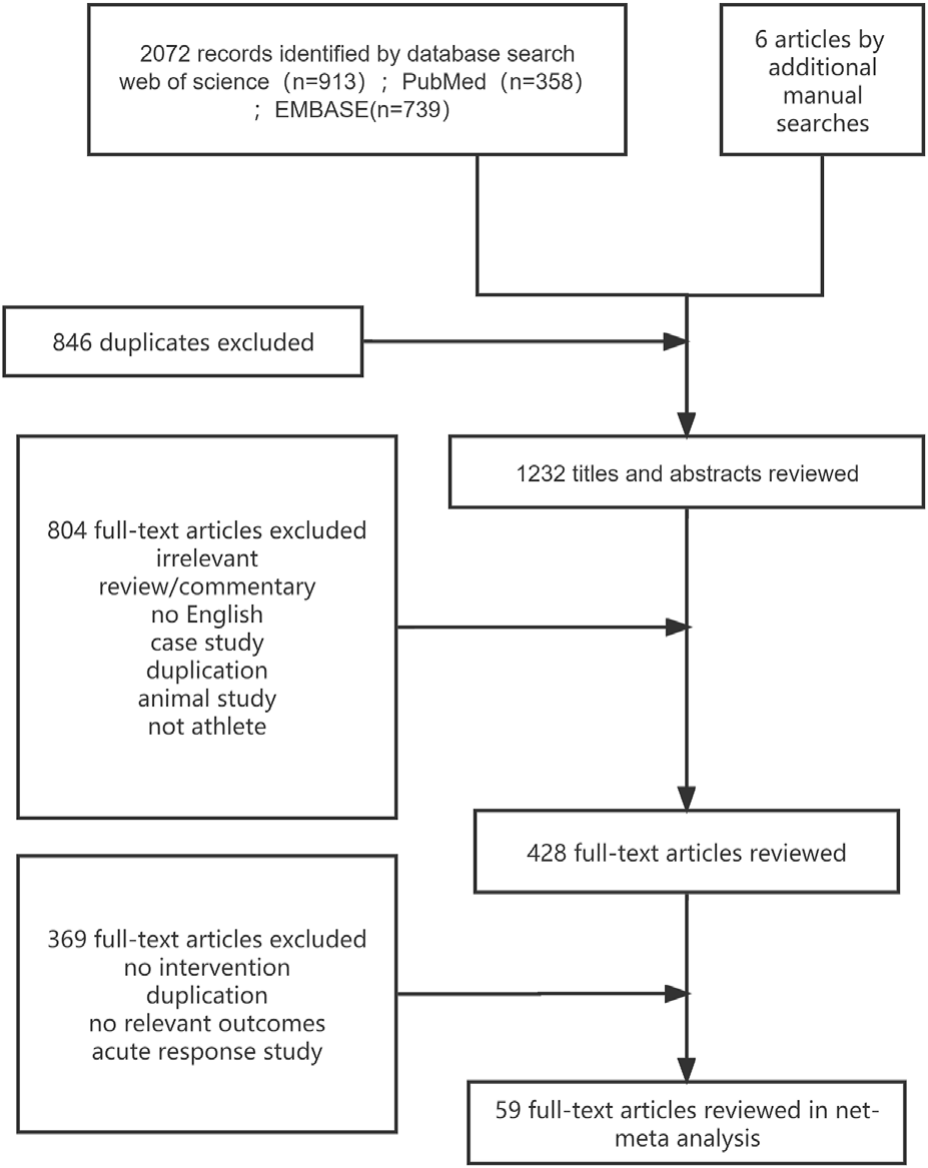
Search terms and outcomes.

A total of 2,072 citations were identified through the search, and 59 eligible articles were retrieved (Figure 1). In the controlled trials that met our inclusion criteria, a total of 1,821 athletes were recruited, with 494 participants assigned to the normoxic training group and 1,327 participants assigned to the altitude/hypoxic training group. The average sample size in respective groups was 9 individuals (range, 5–20), with an average intervention period of approximately 16 days (range, 11–56). In the included trials, male participants were predominant (80.6%), and the vast majority of the included studies recruited endurance athletes (54 studies, 1,175 participants, 91.7%). Further details are provided in Supplementary Appendix S4, Table 4.1. Except for IHE, most of the studies about other methods did not use the blinding method. This phenomenon led to the rating of a high proportion of the included studies as high risk, reaching 46% (see Supplementary Appendix S5 for details).

### Results of the network meta-analysis

**FIGURE 2:**
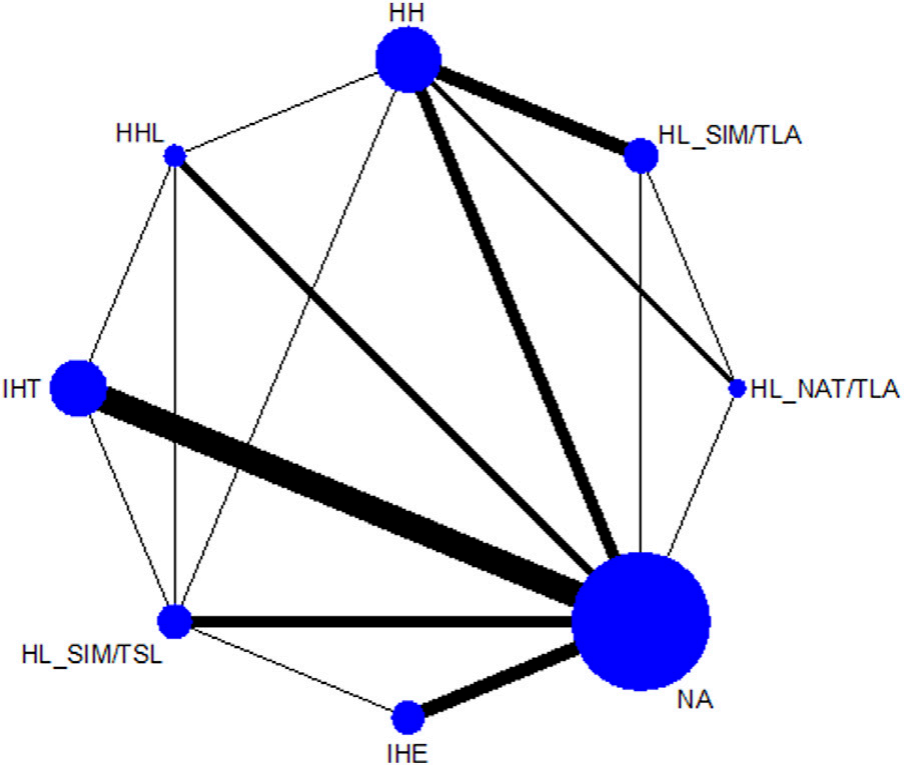
Network graphs for maximal oxygen consumption, and altitude/hypoxic training with direct comparisons are linked with a line; the thickness of connecting lines corresponds to the number of trials evaluating the comparison. HH “training high and living high,” HL_NAT/TLA “living in natural altitude but training at low altitude,” HL_SIM/TLA “training in simulated altitude but training at low altitude,” HL_SIM/TSL “living in a simulated altitude but training at sea level,” IHT “interment hypoxic training,” IHE “interment hypoxic expose,” HHL “living high and training low and high.”

**FIGURE 3:**
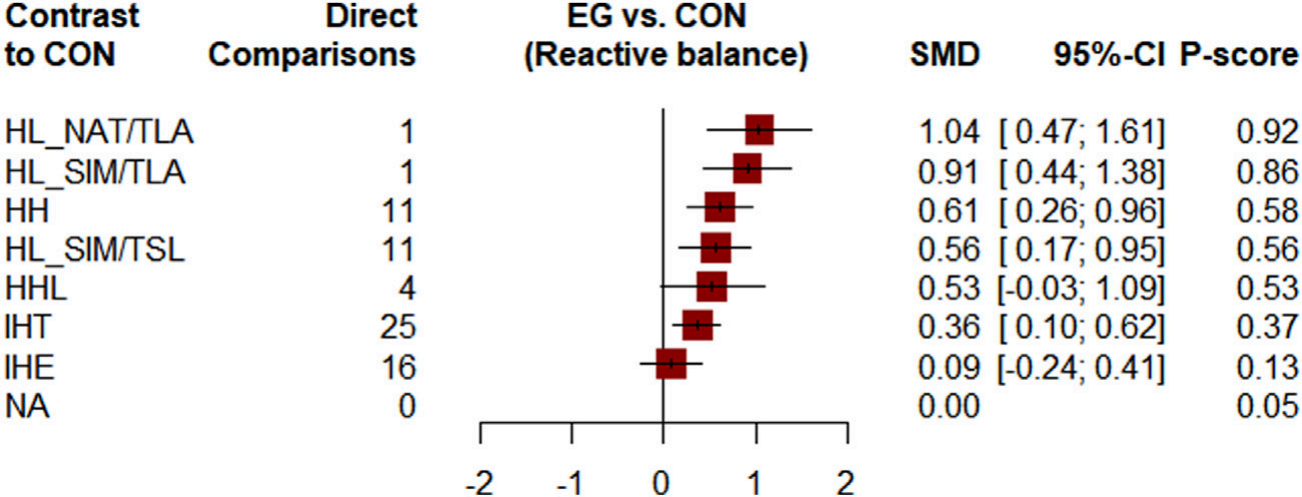
Forest plots for mean differences of altitude/hypoxic training compared with sea level/normoxic training. Confidence of outcomes was graded using the Confidence in Network Meta-Analysis application. HH “training high and living high,” HL_NAT/TLA “living in natural altitude but training at low altitude,” HL_SIM/TLA “training in simulated altitude but training at low altitude,” HL_SIM/TSL “living in a simulated altitude but training at sea level,” IHT “interment hypoxic training,” IHE “interment hypoxic expose,” HHL “living high and training low and high.”

Figure 2 shows the network of eligible comparisons. All AT interventions had at least one normoxic training as a control intervention and were directly compared with at least one other form of AT. The forest plot (Figure 3) shows that all AT interventions, except for IHE and HHL, were significantly more effective than normoxic training in improving athletes’ VO_2_max, with SMDs ranging from 1.04 (95% CrI, 0.47–1.61) for HL_NAT/TLA and 0.36 (95% CrI, 0.10–0.61) for IHT. Based on P-scores, HL combined with low altitude training was the most effective intervention for improving athletes’ VO_2_max (HL_NAT/TLA: 0.92 and HL_SIM/TLA: 0.86) and was significantly better than HL combined with sea level training (0.56). The main results of the network meta-analysis are presented in the league table (Table 1). Regarding improvement of athletes’ VO_2_max, HL_NAT/TLA, HL_SIM/TLA, and HH were superior to IHE (SMDs ranged from 0.52 to 0.96). In addition, the improvement effect of IHT was not as good as that of HL_NAT/TLA (SMD, 0.68; CrI, 0.05–1.30) and HL_SIM/TLA (SMD, 0.55; CrI, 0.01–1.08). The τ value was 0.2089, which was considered small in the context of the observed changes in AT. The I^2^ value was 46.2% (moderate heterogeneity). The global Q score for inconsistency was 14.56, with a *p*-value of 0.6014.

**TABLE 1:** League table for changes in maximal oxygen uptake associated with altitude/hypoxic training. For interpretation, a number larger than zero favours the column-defining treatment of a cell, i.e., this treatment leads to an increase in VO_2max_. Values depicted are mean differences with associated 95% confidence intervals. The upper half is results of direct comparison and the lower half of mixed comparison. HH “training high and living high,” HL_NAT/TLA “living in natural altitude but training at low altitude,” HL_SIM/TLA “training in simulated altitude but training at low altitude,” HL_SIM/TSL “living in a simulated altitude but training at sea level,” IHT “interment hypoxic training,” IHE “interment hypoxic expose,” HHL “living high and training low and high.”

HL_NAT/TLA	−0.01 (−0.82; 0.80)	0.34 (−0.23; 0.90)					**2.73 (1.40; 4.06)**
0.13 (−0.38; 0.64)	HL_SIM/TLA	0.28 (−0.08; 0.64)					0.71 (−0.58; 2.00)
0.43 (−0.04; 0.91)	0.30 (−0.03; 0.64)	HH	0.23 (−1.14; 1.59)	−0.04 (−0.86; 0.77)			**0.56 (0.19; 0.93)**
0.48 (−0.20; 1.16)	0.35 (−0.25; 0.95)	0.05 (−0.46; 0.55)	HL_SIM/TSL	−0.65 (−1.97; 0.67)	0.09 (−0.80; 0.99)	−0.25 (−1.57; 1.06)	**0.67 (0.25; 1.08)**
0.51 (−0.24; 1.26)	0.38 (−0.30; 1.06)	0.08 (−0.52; 0.68)	0.03 (−0.63; 0.69)	HHL	0.65 (−0.65; 1.95)		0.29 (−0.34; 0.92)
**0.68 (0.05; 1.30)**	**0.55 (0.01; 1.08)**	0.24 (−0.19; 0.68)	0.20 (−0.25; 0.65)	0.17 (−0.44; 0.77)	IHT		**0.37 (0.11; 0.64)**
**0.96 (0.30; 1.61)**	**0.83 (0.25; 1.40)**	**0.52 (0.04; 1.00)**	0.48 (−0.02; 0.97)	0.45 (−0.20; 1.09)	0.28 (−0.14; 0.69)	IHE	0.07 (-0.26; 0.40)
**1.04 (0.47; 1.61)**	**0.91 (0.44; 1.38)**	**0.61 (0.26; 0.96)**	**0.56 (0.17; 0.95)**	0.53 (−0.03; 1.09)	**0.36 (0.10; 0.62)**	0.09 (−0.24; 0.41)	NA

### Dose-response correlation

**FIGURE 4:**
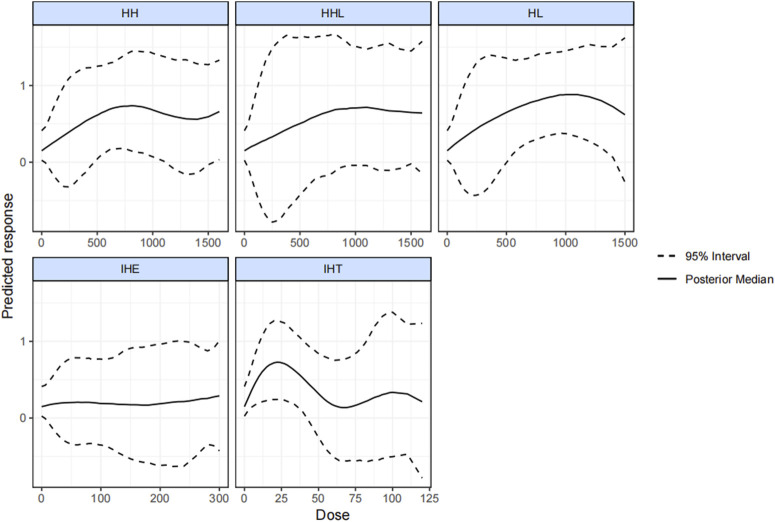
Dose-response associations between different types of altitude training and hypoxia dosage and change in VO_2max_. HH “training high and living high,” HL “living high but living low,” IHT “interment hypoxic training,” IHE “interment hypoxic expose,” HHL “living high and training low and high.”

Figure 4 plots the dose-effect curves for the wide variety of interventions used in the included studies. An inverted u-shaped curve relationship between hypoxic dose and VO_2_max was identified for IHT, HH, and HL. A reasonable hypoxic dose range of HH (from 470 kmh to 1,130 kmh) led to a notable increase of the VO_2_max, and the reasonable dose for HL ranged from 500 kmh to 1,415 kmh. The effectiveness of IHT was no longer significant when the hypoxic dosage exceeded 42.5 kmh. Lastly, we found that HHL was not significantly correlated with IHE.

## Discussion

In this study, the effect of varying hypoxic methods on athlete’s VO_2_max was comprehensively compared and ranked, and the reasonable hypoxic doses range for the respective form was explored. In general, the results showed that HL, HH, and IHT, not IHE and HHL, significantly outperformed normoxic training in enhancing VO_2_max. HL with low altitude training was the optimal effective method (natural HL slightly better than simulated HL), whereas the effects of IHT reached the minimum. Besides, as indicated by the result, the hypoxic dosage of HL, HH, and IHT displayed a U-shaped relationship with a change in VO_2_max. The effective dosage for HH ranged from 470 kmh to 1,130 kmh. For HL, the effective dosage was from 500 kmh to 1,415 kmh. Furthermore, for IHT, the significant effect disappeared when the dosage exceeded 42.5 kmh.

In this study, the efficacy of HL in enhancing aerobic performance at sea level was confirmed (Fulco et al., 2000; Bonetti and Hopkins, 2009), and the reasonable range of hypoxic dosages was determined [a U-shaped relationship was found between the change in VO_2_max and hypoxic dosages (500–1,415 kmh)]. Despite variations in selected indicators, the effective hypoxic dosage in previous studies aligns closely with the reasonable range explored in this study (Rusko et al., 2004; Wilber, 2007; Gore et al., 2013). The U-shaped curve and upper limit of the reasonable dosage are novel findings that may arise from the adverse effects exerted by prolonged chronic hypoxia such as disrupted sleep quality (Kinsman et al., 2005; Sargent et al., 2013), oxidative disorders or pulmonary edema (Gaur et al., 2021; Rytz et al., 2022), accumulated fatigue (Bergeron et al., 2012; Koehle et al., 2014), etc.

Moreover, the effect of training site altitude and type of hypoxic residential environment on the efficacy of HL was investigated. The results showed that the altitude of the training location is the primary determinant of the efficacy of HL, whereas the type of hypoxia in a residential environment has little impact on the outcomes. Extensive research has suggested that the discernible variations in effective HL camps with different training heights are primarily attributed to two underlying factors. 1) Differences in total hypoxic dose: compared with training at sea level, training at low altitudes can provide additional hypoxic exposure (low degree) accompanied with an increase in hypoxic dose, this appropriate dose escalation is capable of inducing greater physiological adaptations accompanied by improvements in blood oxygen carrying capacity and VO_2_max and 2) the dual stimulation of environmental and exercise hypoxia: exposure to low altitudes compared with exposure to sea level altitudes can provide moderate hypoxic stress relative, such that appropriate metabolic and neural adaptations can be stimulated (without excessive damage to training quality such as training in moderate altitudes) (Zoll et al., 2006; Rodríguez et al., 2015). The findings of the influence of training altitude on the intervention efficacy of HL camps are highly relevant to the selection of an altitude base. No marked difference in chronic adaptive changes between simulated HL (HL_SIM/TAL) and natural HL (HL_NAT/TAL) was found when the altitude of the training site was similar. This observation is supported by several previous studies (Hauser et al., 2016; Saugy et al., 2016; Bourdillon et al., 2017), although athletes exhibit varying physiological reactions to acute normobaric and hypobaric hypoxia exposure (Loeppky et al., 1997; Girard et al., 2012; Beidleman et al., 2014; Coppel et al., 2015). In practical applications, simulating HL appears to be a more favorable method due to lower adverse impact risks (Saugy et al., 2016), economic and time costs (Carr et al., 2019; Millet et al., 2010), and more flexible operations (Millet et al., 2010).

Although HL has become the dominant training method in academic circles, classic HH remains a preferred choice among many elite athletes worldwide (Álvarez-Herms et al., 2015). This study indicates that HH camps with a recommended dosage range of 470–1,130 kmh can significantly improve athletes’ VO_2_max compared with that observed with normoxic training. Regarding effectiveness, HH ranked lower than HL with low altitude training. The above-mentioned observations may be explained by previous reports that the critical limitation of HH relative to HL is the heightened hypoxic stress at moderate altitudes (Levine and Stray-Gundersen, 1997; Carr et al., 2015), which can impede the generation of necessary training intensities for eliciting beneficial adaptations (Wehrlin and Hallén, 2006). Furthermore, except for the interference of various confounding factors, the lower upper limit and wider CI for the dose-response curve may correlate with an additional drawback of HH; the risk of adverse reactions in an HH condition is higher than in an HL condition due to greater hypoxic continuity (Brocherie et al., 2017; Mujika et al., 2018). Thus, to minimize this potential risk and maximize the benefits of AT, an effective health and load monitoring system should be developed (Sperlich et al., 2016; Schmitt et al., 2018; Mujika et al., 2019) and adequate nutritional support should be provided (Govus et al., 2015; Stellingwerff et al., 2019). Currently, the investigated lower limit of the hypoxic dose of HH is lower than the previous study results (Millet et al., 2010). Our findings indicate that athletes who partake in 2–3 weeks of low altitude camps can effectively enhance their VO_2_max (Gore et al., 1997; Carr et al., 2015). Compared with moderate and sea level altitudes, low altitude provides less hypoxic stimulation but maintains higher training quality and also offers dual stimulation of hypoxia and training. This makes low altitude camping a valuable adaptive measure for athletes preparing for higher altitude training or competitions (Saunders et al., 2009). Lastly, it is worth noting that HL with sea level training had effects similar to those of HH, which has also been reported in a similar study by Carr et al. (2019). The above perspective shows that passive hypoxia exposure alone may not maximize performance gains.

It is currently understood that the noteworthy enhancement of VO_2_max following HH or HL stems predominantly from hematological adaptation following chronic hypoxia exposure, which increases the total amount of hemoglobin accompanied by the enhancement of oxygen-carrying capacity (Hahn and Gore, 2001). Multiple studies have presented compelling evidence on aerobic adaptation under hypoxic conditions, with molecular mechanisms underlying the above-described hematological changes being elucidated. Furthermore, multiple studies have presented molecular-level evidence supporting these alterations in hematology such as hypoxia-inducible factor-1 (HIF-1), which is vital to the transcriptional regulation of erythropoietin (Bhattarai et al., 2018; Koyasu et al., 2018). However, Saunders et al.’s study shows a correlation between VO_2_max and hemoglobin mass changes with an *r* ^2^ of only 0.15, suggesting that factors beyond hematological indices may account for 85% of the variation in VO_2_max (P. U. Saunders et al., 2013). Besides activating the EPO transcription gene, HIF-1 is also required for inducing other genes (e.g., encoding glycolytic enzymes, VEGF, GLUT-1, and metabolic proteins), which causes molecular changes and triggers non-hematological physiological adaptations such as increased citrate synthase activity, mitochondrial density, capillary-to-fiber ratio, and muscle buffering capacity (Wang et al., 1995; Vogt et al., 2001; Gore et al., 2007). The above changes may partially account for the improvement in VO_2_max independent of hematological adaptions.

This study confirmed the positive effect of IHT on athletes’ VO_2_max, which is consistent with a recent meta-analysis (Westmacott et al., 2022). Besides, we found two novel observations: 1) the minimum effect by IHT and 2) the reasonable hypoxic dosage range. First, the effectiveness of IHT may arise from its specific underlying mechanism or methodological design (Czuba, Fidos-Czuba et al., 2018). Unlike HH and HL, numerous studies have indicated that the hypoxic exposure of IHT is inadequate to substantially alter hematological parameters (Czuba et al., 2014; Czuba et al., 2019; Ambroży et al., 2020; Kim et al., 2021; Park et al., 2022; Teległów et al., 2022), and the potential mechanism for enhancing aerobic capacity primarily involves specific molecular adaptations in peripheral tissues (Ponsot et al., 2006; Zoll et al., 2006; Neya et al., 2007). It is worth noting that Czuba et al. posited that a hematological mechanism is more effective in enhancing aerobic capacity (Czuba et al., 2018), and the diverse adaptations due to IHT contributes greatly to exercise (Green et al., 2000; Hamlin et al., 2010; Park et al., 2016; Saunders et al., 2004), which can also serve as a valuable metric for evaluating aerobic performance (Levine and Stray-Gundersen, 2005). In summary, the physiological adaptations induced by IHT are unable to increase VO_2_max as significantly as that observed with HH and HL.

Although this study has explored a relatively robust dose-effect model for IHT, we found, after reviewing the included studies, that the model is unreasonable due to the oversight of a crucial indicator, training intensity under hypoxia, which is essential for promoting muscle and metabolic adaptations (McLean et al., 2014). Unfortunately, it is difficult to standardize the specific training intensity in each study and simultaneously consider the combined effects of hypoxic and training loads. Notably, several studies have stated that IHT can enhance athletes’ anaerobic capacity (Hendriksen and Meeuwsen, 2003; Millet et al., 2014; Morton and Cable, 2005), which is to an extent significant in team and high-level endurance sports. In summary, although IHT had the least impact on VO_2_max, it considerably important in practice due to the low cost and low risk of negative effects (Jung et al., 2020).

Theoretically, variant combining HHL with HL and LLTH should produce positive effects (Millet et al., 2010). However, our finding was not consistent with the theoretical expectation. Possible reasons include limited quantity of relevant experiments performed in the included studies, small sample sizes, and low statistical power. Furthermore, as revealed by most included studies, HHL can directly enhance time-trial performance (Robertson et al., 2010; Czuba et al., 2014), and some studies have reported no significant correlation between performance enhancement and changes in blood oxygen-carrying capacity (Czuba et al., 2014; Rodríguez et al., 2015). Although using VO_2_max as the sole evaluation criterion for HHL is insufficient, the lack in both the depth and breadth of research makes it difficult to comprehensively assess the effect of HHL. In-depth research should prioritize exploring HL to identify the most effective combination with other training methods.

The effectiveness of IHE in augmenting the aerobic performance of athletes has become a contentious topic (Millet et al., 2010; Viscor et al., 2018), with detractors contending that IHE fails to elicit significant physiological adaptations (Brugniaux et al., 2006; Rodríguez et al., 2007). For physiological or kinematic indicators, IHE failed to demonstrate any substantial improvement as indicated by over half of the included studies (6/9). Compared with other practices, IHE employs an extremely lower overall hypoxic dose and lacks supplementary high-intensity training stimuli during hypoxia exposure. This relatively limited and inadequate stimulation of athletes makes the feasibility of IHE questionable, and the driving force behind the development of this method may be more commercial than scientific (Lundby and Robach, 2016).

## Limititation

The absence of blinding in most existing studies posed a high risk of bias in quality assessment and undermines the ability to rule out placebo effects. A cross-over design can be employed although blinding may not be feasible for AT in natural environments. Future relevant studies should adopt more rigorous experimental designs to improve the evidence on effectiveness and dosage accuracy. The efficacy of AT is not solely determined by the degree of hypoxia and exposure continuity, as various confounding factors that affect the final outcome have not been fully considered. These factors include the specific training content, load and intensity in hypoxic or normoxic environments, individual differences in hypoxic responses, and the criticality of various support work or specialty-specific factors (Burtscher et al., 2018). Unfortunately, quantifying the above-described variables is challenging and may increase the heterogeneity risk in this study.

## Conclusion

The findings of this comprehensive network meta-analysis suggest that HL, HH, and IHT compared with normoxic training are capable of effectively improving the VO_2_max of athletes. The effect of HL with low altitude training was optimal, and the hypoxic type of residential environments (simulated or natural) did not affect the results. This observation can provide a useful reference for future site selection of an altitude base. In addition, this study also found a dose-effect relationship between hypoxic dose and VO_2_max variation for both HH and HL. As indicated by the results, hypoxic practices have maximum and minimum effective hypoxic dose thresholds, and both showed an inverted U-shaped curve between dose and efficacy. This observation may be correlated with the negative effects of chronic hypoxia, and it also provides sound recommendations regarding the time and height arrangements for altitude camps. Notably, as opposed to the previous study findings, HH camps organized at low altitudes can moderately enhance the VO_2_max of athletes, suggesting that it can be a valuable adaptive measure for athletes preparing for higher AT or competitions. The small number of studies on HHL in this meta-analysis limits the confidence of our findings. Following a brief review of other indicators, the combined approach seems to be optional, and the depth and breadth of relevant explorations should be improved in the future. Lastly, the quality of most studies was low. To maximize the exclusion of placebo effects, future controlled trials on simulated altitudes should consider using a blinded method, and controlled trials on real altitudes can attempt using the cross-over design.
