# Supplementary material for: Optimal type and dose of hypoxic training for improving maximal aerobic capacity in athletes: a systematic review and Bayesian model-based network meta-analysis
Source: Front Physiol. 2023 Sep 5;14:1223037. doi: 10.3389/fphys.2023.1223037 (PMC10513096; doi:10.3389/fphys.2023.1223037)
Supplement: Supplementary file 1 [file Table1.docx]

**Optimal Hipoxia Dose and Type of altitude training to Improve Maximum aerobic capacity in athletes : A Systematic Review and Bayesian Model-Based Network Meta-Analysis**

**Supplementary file 1: Intervention coding and Dataset**

1. **Intervention coding**

This supplementary file shows the Intervention coding:this study categorizes the included altitude/hypoxic training methods into three levels: (1) encoding based on the methodological design of altitude/hypoxic training, including "HH" (live-high train-high), "HL" (live-high train-low), "IHT" (intermittent hypoxic training), "IHE" (intermittent hypoxic exposure) and "HHL" (live high–train low and high); (2) Subsequently, based on the first-level encoding, we further refined the HL’s categorization according to the type of hypoxia in residential environment and the altitude of training location as follows:including "HL_NAT/TLA" (living in a real altitude(hypobaric hypoxia) and training at low altitude(1000m-1500m)), "HL_SIM/TLA" (training in a simulated altitude(normobaric hypoxia) and training at low altitude (1000m-1500m)), and "HL_SIM/TSL" (living in a simulated altitude(normobaric hypoxia) and training near sea level (<600m)). In addition, we expanded the altitude range of the HH and included some control groups (which are not usually explicitly defined as HH in research) that underwent experiments at low altitudes into the category of HH.We will use a random-effects network meta-analysis within a Bayesian framework to rank and evaluate the intervention effects of various measures in this level. (3) Finally, the third level of encoding is also based on the first level, where the intervention measures are encoded at the intersection of specific types and dosages, and this level will be applied in random-effects Bayesian Model-Based Network MetaAnalysis (MBNMA).

1. **Dataset**

This supplementary file shows the datasets（i.e., at altitude/hypoxia training hypoxia dose level) used in this study. The *studyID* indicates the name of the author and the year of publication. The *intervention* indicates the specific training methods. The *exact_dose* parameter indicates the exact hypoxic exposure estimated per week that participants accumulated in the study. The *approximation*_*dose* indicates the group of doses by approximation. The *residual_dose* indicates the difference between the exact dose and the dose allocated by approximation. The *Heigh (m /%)* refers to the altitude where the subject is actually exposed, with "/", to represent training and life.The *Exposure / intervention days* is the days of participation in the activities and the frequency of the training sessions.The *Hypoxic (h / d or h / session)* is the duration of hypoxia exposure, daily or per session.*The total hypoxic exposure* is the total hypoxia exposure duration of the subjects throughout the experimental session.HH “training high and living high”; HL “living high but living low”; IHT “interment hypoxic training”; IHE “interment hypoxic expose”;HHL “living high and training low and high”.

| studyID | agent | exact_dose | approximation_  dose | residual_dose | Heigh(m/%) | Exposure/  intervention  days | Hypoxic  (h/d or h/session) | Total hypoxic exposure |
| --- | --- | --- | --- | --- | --- | --- | --- | --- |
| Benjamin  2018 | HH | 1680 | 1650 | 30 | 2500m | 28 | 24 | 672 |
|  | HL | 1534 | 1500 | 34 | 1250m/2500m | 28 | 4/20 | 672 |
| Eileen Y. Robertson  2010 | HHL | 915 | 900 | 15 | 2200m/3000m | 21  （4 session/week） | 1.25/14 | 309 |
|  | HL | 882 | 900 | 18 | 3000m | 21 | 14 | 294 |
| Ferran  2015 | HH | 1169 | 1200 | 31 | 2320m | 21 | 24 | 504 |
|  | HH | 1559 | 1500 | 59 |  | 28 | 24 | 672 |
|  | HHL | 1559 | 1500 | 59 | 690m/2320m | 28 | 24 | 672 |
| Kazunobu Okazaki  2019 | HH | 1680 | 1650 | 30 | 2500m | 28 | 24 | 672 |
| Zhijun Meng  2021 | HH | 3050 | 1650 | 1400 | 2280m | 56 | 24 | 1334 |
| C.]. Core  1997 | HH | 1169 | 1200 | 31 | 1740m | 28 | 24 | 672 |
|  | HH | 873.6 | 900 | 26.4 | 1300m | 28 | 24 | 672 |
| M. Burtscher  1996 | HH | 667 | 650 | 17 | 2315m | 12 | 24 | 288 |
| Amelia J. Carr  2018 | HH | 464 | 500 | 36 | 1380m | 14 | 24 | 336 |
|  | HL | 588 | 600 | 12 | 3000m | 14 | 14 | 196 |
| Hun-Young Park  2019 | HL | 756 | 750 | 6 | 3000m | 21 | 12 | 252 |
| Czuba M  2018 | HL | 507.15 | 500 | 7.15 | 2100m | 21 | 11.5 | 241.5 |
|  | IHT | 11 | 10 | 1 | 2100m | 21  (3 session/week) | 35min | 5.25 |
| Paul Robach  2015 | HL | 1484 | 1500 | 16 | 1100m/3000m | 28 | 10/14 | 672 |
|  | HH | 739.2 | 750 | 10.8 | 1100m | 28 | 24 | 672 |
| Clare E. Humberstone-Gough  2013 | HL | 714 | 700 | 14 | 3000m | 17 | 14 | 238 |
|  | IHE | 48.45 | 50 | 11.55 | 4750m | 17  (1 session/day) | 36min | 10.2 |
| C.J.GORE  2001 | HL | 656 | 650 | 6 | 3000m | 23 | 9.5 | 218.5 |
| EILEEN Y. ROBERTSON  2010 | HL | 882 | 900 | 18 | 3000m | 21 | 14 | 294 |
| P.U. Saunders  2010 | HL | 882 | 900 | 18 | 3000m | 21 | 14 | 294 |
| P.U. Saunders  2009 | HL | 1092 | 1100 | 8 | 2860m | 56  (5 sessions/week) | 9 | 360 |
| Laurent Schmitt  2018 | HL | 740 | 750 | 10 | 1150m/2700m | 15 | 10/14 | 360 |
|  | HH | 414 | 400 | 14 | 1150m | 15 | 24 | 360 |
| Amelia J. Carr  2015 | HH | 696 | 650 | 46 | 1380m | 21 | 24 | 504 |
|  | HL | 1002 | 1100 | 98 | 1380m/3000m | 21 | 15/9 | 504 |
| Dariusz Sitkowski  2019 | HL | 785 | 750 | 35 | 1100m/2200m | 21 | 11/13 | 504 |
|  | Placebo(HH) | 554 | 500 | 54 | 1100m | 21 | 24 | 504 |
|  | HH | 453.6 | 400 | 53.6 | 900m | 21 | 24 | 504 |
| Christoph Siebenmann  2011 | HL | 1484 | 1500 | 16 | 1200m/3000m | 28 | 10/14 | 672 |
|  | HH | 806 | 750 | 56 | 1200m | 28 | 24 | 672 |
| Mitsuo Neya  2013 | HL | 1056 | 1250 | 194 | 1300m/3000m | 22 | 14/10 | 528 |
|  | HH | 686 | 700 | 14 | 1300m | 22 | 24 | 528 |
| L. Schmitt  2008 | HL | 875 | 900 | 25 | 1200m/3000m | 18 | 13/11 | 432 |
|  | HH | 518 | 500 | 18 | 1200m | 18 | 24 | 432 |
| L. Schmitt  2006 | HL | 940 | 900 | 40 | 1200m/3000m | 18 | 11/13 | 432 |
|  | HH | 518 | 500 | 18 | 1200m | 18 | 24 | 432 |
| Julien V. Brugniaux  2005 | HL | 922 | 900 | 22 | 1200m/2800m | 18 | 10/14 | 432 |
|  | HH | 518 | 500 | 18 | 1200m | 18 | 24 | 432 |
| Paul Robach  2017 | HL | 1164 | 1100 | 64 | 1035m/2207m | 26 | 7/17 | 624 |
|  | HH | 646 | 650 | 4 | 1035m | 26 | 24 | 624 |
| C. Dehnert  2002 | HL | 487 | 500 | 13 | 800m/2000m | 14 | 11/13 | 336 |
|  | HH | 269 | 250 | 16 | 800m | 14 | 24 | 336 |
| Milosz Czuba  2014 | HHL | 1008 | 1000 | 8 | 3000m/1000m/2015m | 21 | 24 | 504(19/30/455) |
| Hun-Young Park  2018 | IHT | 81 | 80 | 1 | 3000m | 42  (3 sessions/week) | 1.5 | 27 |
| PREETIWAT WONNABUSSAPAWICH  2017 | HHL | 1109 | 1100 | 9 | 825m/3000m | 56  (3 sessions/week) | 0.25 | 1134 |
| Miłosz Czuba  2013 | IHT | 12.15 | 15 | 2.85 | 2500m | 21  (6 sessions/week) | 16min | 4.86 |
| Won-Sang Jung  2020 | IHT | 54 | 55 | 1 | 3000m | 42  (3 sessions/week) | 1 | 18 |
| Hun-Young Park  2022 | IHT | 54 | 55 | 1 | 3000m | 42  (3 sessions/week) | 1 | 18 |
| Gre´goire Millet  2014 | IHT | 57.3 | 55 | 2.3 | 3000m | 21  (2 sessions/week) | - | 19.1 |
| Milosz Czuba  2011 | IHT | 13.9 | 15 | 1.1 | 2650m | 21  (3 sessions/week) | 35min | 5.25 |
| BEN A. HOLLISS  2014 | IHT | 22.9 | 25 | 2.1 | 2150m | 56  (2 sessions/week) | 40min | 10.7 |
| Miłosz Czuba  2017 | IHT | 16.7 | 16.7 | 1.7 | 2500m | 28  (2 sessions/week) | 50min | 6.7 |
| Ingrid J.M. Hendriksen  2003 | IHT | 43.75 | 45 | 1.25 | 2500m | 10  (1 sessions/day) | 105min | 17.5 |
| DJ Ramos-Campo  2015 | IHT | 54.25 | 55 | 0.75 | 3875m（14.5%-15%） | 49  (2 sessions/week) | 1 | 14 |
| M. J. TRUIJENS  2002 | IHT | 20 | 20 | 0 | 3530m（15.3%） | 35  (3 sessions/week) | 22.75min | 5.68 |
| Sung-Woo Kim  2019 | IHT | 81 | 80 | 1 | 3000m | 42  (3 sessions/week) | 1.5 | 27 |
| Aneta Teległów  2022 | IHT | 21.6 | 20 | 1.6 | 2400 | 21  (3 sessions/week) | 1 | 9 |
| Damon Arezzolo  2020 | IHT | 29 | 30 | 1 | 3625(14.7%) | 28  (2 sessions/week) | 1 | 8 |
| Elodie Ponsot  2005 | IHT | 24 | 25 | 1 | 3000m | 42  (2 sessions/week) | 40min | 8 |
| Ste´phane P. Dufour  2006 | IHT | 24 | 25 | 1 | 3000m | 42  (2 sessions/week) | 40min | 8 |
| James Peter Morton  2014 | IHT | 33 | 35 | 2 | 2750m | 56  (3 sessions/week) | 30min | 12 |
| Tadeusz Ambrozy  2020 | IHT | 120 | 120 | 0 | 4000m | 42  (5 sessions/week) | 1 | 30 |
| Colleen G. Julian  2003 | IHE | 82.5 | 85 | 2.5 | 6875m（11%） | 56  (3 sessions/week) | 0.5 | 12 |
| M. Burtscher  2010 | IHE | 130.5 | 130 | 0.5 | 4350m | 35  (3 sessions/week) | 2 | 30 |
| Ferran A. Rodríguez  2014 | IHE | 285 | 285 | 0 | 4750m | 28  (5 sessions/week) | 3 | 60 |
| Keisho Katayama  2004 | IHE | 226.8 | 250 | 23.2 | 5400m | 14  (1 sessions/day) | 3 | 42 |
| Darrell L. Bonetti  2006 | IHE | 103.1 | 100 | 3.1 | 6875m（11%） | 21  (5 sessions/week) | 1 | 15 |
| Martin J. Truijens  2014 | IHE | 285 | 285 | 0 | 4750m | 28  (5 sessions/week) | 3 | 60 |
| ANDREW E. KILDING  2016 | IHE | 22.5 | 25 | 2.5 | 3000m | 15  (1 sessions/day) | 0.5 | 7.5 |
| VAHID TADIBI  2007 | IHE | 82.5 | 85 | 2.5 | 5500m | 15  (1 sessions/day) | 1 | 15 |
| KEISHO KATAYAMA  2003 | IHE | 60.75 | 60 | 0.75 | 4500m | 21  (3 sessions/week) | 1.5 | 13.5 |

**Supplementary File 2: Assessment of Connectivity, Consistency and Transitivity in Network Meta DoseResponse Analysis**

There are three key assumptions to conduct a Network Meta DoseResponse Analysis: (1) network connectivity, (2) consistency in the data, and (3) transitivity.

**(1)Connectivity**

Connectivity is a key assumption in network meta-dose analysis, and evidence of unconnectedness may lead to low statistical power and misleading results (Ter Veer, van Oijen, & van Laarhoven, 2019). Our results show that there is no phenomenon of poor connectivity (Figures 1.1,2)


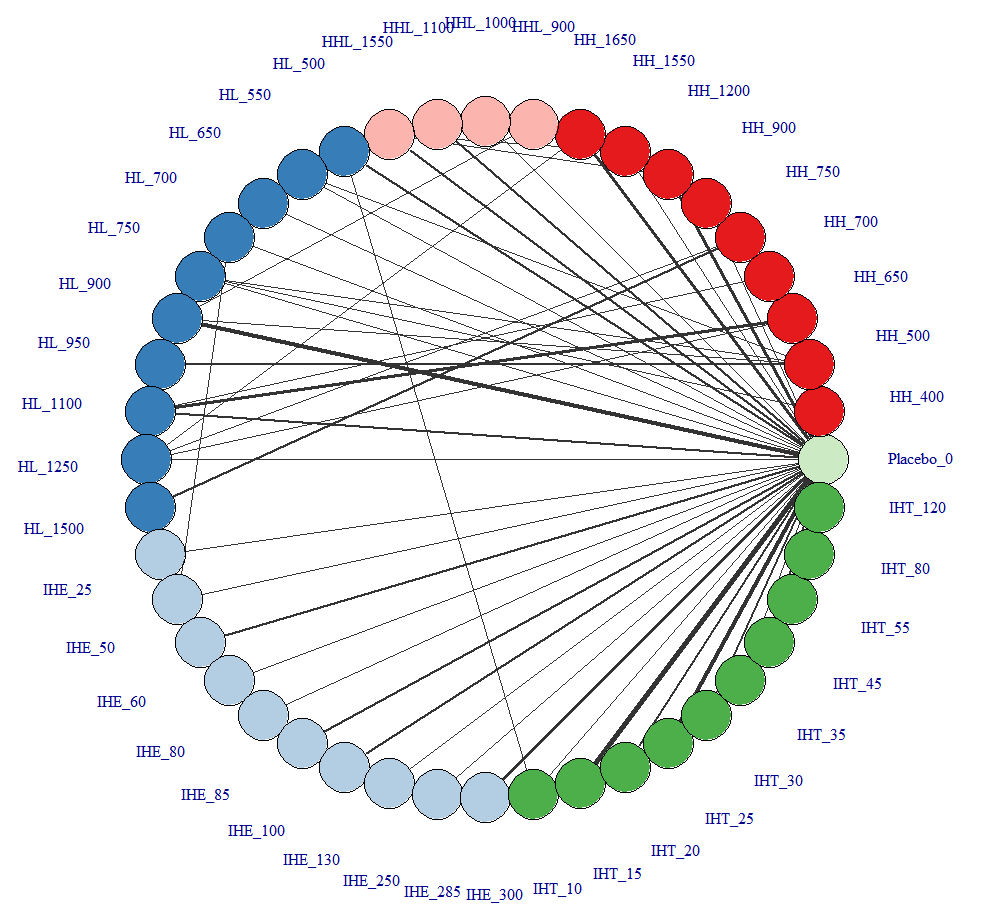


**Supplementary Figures 2.1.** Treatment-level network. The first value indicates the specific altitude/hypoxic training and the second one is the corresponding dose of that intervention. HH “training high and living high”; HL “living high but living low”; IHT “interment hypoxic training”; IHE “interment hypoxic expose”;HHL “living high and training low and high”.


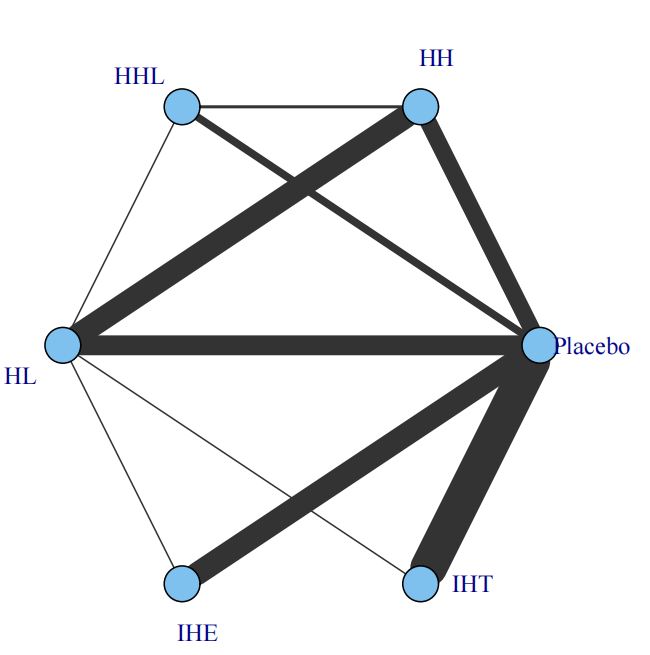


**Supplementary Figure 2.2.** Agent-level network. The first value indicates the specific altitude/hypoxic training and the second one is the corresponding dose of that intervention. HH “training high and living high”; HL “living high but living low”; IHT “interment hypoxic training”; IHE “interment hypoxic expose”;HHL “living high and training low and high”.

**(2)Consistency**

We analyzed the data with the consistency model and the unrelated mean effect model, and compared the differences in the deviation, the number of estimated parameters in the network, and the Deviance Informative Criterion (DIC) indicators of the two models. If these are similar, it means that our research has good consistency (Wheeler, Hickson, & Waller, 2010). Comparison of these parameters indicated good consistency across models (Table 1.1).

**Supplementary Table 2.1.** Consistent and UME models fit comparison

| Model | Pd | Residual deviance | Deviance | DIC | SD |
| --- | --- | --- | --- | --- | --- |
| Consistent | 146.3 | 156.042 | 341.600 | 486.9 | 0.820 |
| UME | 134.5 | 155.067 | 340.624 | 474.1 | 0.751 |

pD: Number of estimated parameters; DIC: Deviance Informative Criterion; SD: Standard Deviation; UME: Unrelated Mean Effects. Scientific literature indicated that the main indicator to assess the model fit is the DIC. As lower DIC, better fit.

**(3)Transitivity**

We assessed transitivity via MBNMA node-splitting approach. This method splits and compares contributions for a particular treatment contrast into direct and indirect evidence (van Valkenhoef, Dias, Ades, & Welton, 2016). Similar effects denote good transitivity. Figures 1.3 (density plots) below present the results for transitivity in this meta-analysis.

========================================

Node-splitting analysis of inconsistency

========================================

|Comparison | p-value| Median| 2.5%| 97.5%|

|:---------------------|-------:|------:|------:|-----:|

|HL_900 vs HHL_900 | 0.416| | | |

|-> direct | | -0.617| -2.322| 1.167|

|-> indirect | | 0.226| -0.491| 0.928|

|-> MBNMA | | 0.107| -0.565| 0.770|

| | | | | |

|HL_1500 vs HH_750 | 0.493| | | |

|-> direct | | 0.005| -1.170| 1.247|

|-> indirect | | 0.155| -0.243| 0.574|

|-> MBNMA | | 0.138| -0.234| 0.531|

| | | | | |

|HL_1250 vs HH_750 | 0.353| | | |

|-> direct | | -0.136| -1.950| 1.614|

|-> indirect | | 0.156| -0.227| 0.535|

|-> MBNMA | | 0.138| -0.234| 0.526|

| | | | | |

|HL_1100 vs HH_700 | 0.369| | | |

|-> direct | | 0.020| -1.676| 1.792|

|-> indirect | | 0.139| -0.259| 0.537|

|-> MBNMA | | 0.137| -0.234| 0.519|

| | | | | |

|HL_1250 vs HH_650 | 0.366| | | |

|-> direct | | -0.146| -1.814| 1.584|

|-> indirect | | 0.142| -0.229| 0.533|

|-> MBNMA | | 0.138| -0.231| 0.528|

| | | | | |

|HL_1100 vs HH_650 | 0.575| | | |

|-> direct | | 0.051| -0.979| 1.064|

|-> indirect | | 0.137| -0.241| 0.528|

|-> MBNMA | | 0.137| -0.231| 0.519|

| | | | | |

|HL_950 vs HH_500 | 0.295| | | |

|-> direct | | 0.744| -0.434| 1.908|

|-> indirect | | 0.067| -0.329| 0.442|

|-> MBNMA | | 0.138| -0.231| 0.519|

| | | | | |

|HL_900 vs HH_500 | 0.399| | | |

|-> direct | | 0.319| -1.341| 2.010|

|-> indirect | | 0.126| -0.250| 0.518|

|-> MBNMA | | 0.138| -0.231| 0.519|

| | | | | |

|HL_750 vs HH_500 | 0.333| | | |

|-> direct | | -0.337| -2.165| 1.434|

|-> indirect | | 0.155| -0.211| 0.555|

|-> MBNMA | | 0.137| -0.231| 0.519|

| | | | | |

|HL_750 vs HH_400 | 0.383| | | |

|-> direct | | 0.413| -1.203| 2.066|

|-> indirect | | 0.116| -0.257| 0.493|

|-> MBNMA | | 0.138| -0.231| 0.519|

| | | | | |

|IHT_120 vs Placebo_0 | 0.365| | | |

|-> direct | | 0.086| -1.561| 1.684|

|-> indirect | | 0.453| 0.097| 0.831|

|-> MBNMA | | 0.444| 0.085| 0.796|

| | | | | |

|IHT_80 vs Placebo_0 | 0.515| | | |

|-> direct | | 0.446| -0.751| 1.628|

|-> indirect | | 0.440| 0.059| 0.817|

|-> MBNMA | | 0.441| 0.085| 0.792|

| | | | | |

|IHT_55 vs Placebo_0 | 0.121| | | |

|-> direct | | -0.314| -1.170| 0.486|

|-> indirect | | 0.597| 0.239| 0.982|

|-> MBNMA | | 0.438| 0.085| 0.784|

| | | | | |

|IHT_45 vs Placebo_0 | 0.399| | | |

|-> direct | | -0.010| -1.200| 1.130|

|-> indirect | | 0.478| 0.084| 0.864|

|-> MBNMA | | 0.436| 0.083| 0.779|

| | | | | |

|IHT_35 vs Placebo_0 | 0.306| | | |

|-> direct | | -0.095| -1.844| 1.672|

|-> indirect | | 0.452| 0.110| 0.800|

|-> MBNMA | | 0.432| 0.083| 0.773|

| | | | | |

|IHT_30 vs Placebo_0 | 0.422| | | |

|-> direct | | 0.136| -1.095| 1.342|

|-> indirect | | 0.459| 0.084| 0.801|

|-> MBNMA | | 0.429| 0.081| 0.769|

| | | | | |

|IHT_25 vs Placebo_0 | 0.021| | | |

|-> direct | | 1.607| 0.813| 2.411|

|-> indirect | | 0.223| -0.120| 0.582|

|-> MBNMA | | 0.425| 0.079| 0.757|

| | | | | |

|IHT_20 vs Placebo_0 | 0.339| | | |

|-> direct | | -0.091| -1.323| 1.202|

|-> indirect | | 0.459| 0.087| 0.796|

|-> MBNMA | | 0.420| 0.073| 0.746|

| | | | | |

|IHT_15 vs Placebo_0 | 0.227| | | |

|-> direct | | 0.985| 0.172| 1.757|

|-> indirect | | 0.284| -0.062| 0.647|

|-> MBNMA | | 0.411| 0.068| 0.736|

| | | | | |

|IHT_10 vs Placebo_0 | 0.398| | | |

|-> direct | | 0.672| -0.754| 2.093|

|-> indirect | | 0.381| 0.015| 0.728|

|-> MBNMA | | 0.396| 0.064| 0.716|

| | | | | |

|IHE_300 vs Placebo_0 | 0.570| | | |

|-> direct | | 0.324| -0.685| 1.320|

|-> indirect | | 0.001| -0.501| 0.497|

|-> MBNMA | | 0.063| -0.374| 0.516|

| | | | | |

|IHE_285 vs Placebo_0 | 0.441| | | |

|-> direct | | -0.223| -1.823| 1.486|

|-> indirect | | 0.086| -0.374| 0.575|

|-> MBNMA | | 0.063| -0.373| 0.514|

| | | | | |

|IHE_250 vs Placebo_0 | 0.413| | | |

|-> direct | | 0.182| -1.505| 1.958|

|-> indirect | | 0.058| -0.354| 0.503|

|-> MBNMA | | 0.063| -0.372| 0.511|

| | | | | |

|IHE_130 vs Placebo_0 | 0.512| | | |

|-> direct | | -0.108| -1.456| 1.241|

|-> indirect | | 0.077| -0.391| 0.532|

|-> MBNMA | | 0.061| -0.366| 0.504|

| | | | | |

|IHE_100 vs Placebo_0 | 0.505| | | |

|-> direct | | 0.079| -1.292| 1.454|

|-> indirect | | 0.056| -0.403| 0.510|

|-> MBNMA | | 0.060| -0.362| 0.503|

| | | | | |

|IHE_85 vs Placebo_0 | 0.422| | | |

|-> direct | | 0.227| -1.472| 1.865|

|-> indirect | | 0.050| -0.383| 0.483|

|-> MBNMA | | 0.059| -0.360| 0.500|

| | | | | |

|IHE_80 vs Placebo_0 | 0.235| | | |

|-> direct | | -0.931| -2.703| 0.776|

|-> indirect | | 0.133| -0.310| 0.552|

|-> MBNMA | | 0.059| -0.360| 0.500|

| | | | | |

|IHE_60 vs Placebo_0 | 0.491| | | |

|-> direct | | 0.201| -1.090| 1.495|

|-> indirect | | 0.044| -0.369| 0.497|

|-> MBNMA | | 0.058| -0.359| 0.499|

| | | | | |

|IHE_50 vs Placebo_0 | 0.315| | | |

|-> direct | | 0.746| -0.759| 2.306|

|-> indirect | | -0.009| -0.458| 0.421|

|-> MBNMA | | 0.058| -0.358| 0.498|

| | | | | |

|IHE_25 vs Placebo_0 | 0.292| | | |

|-> direct | | -0.685| -2.475| 1.111|

|-> indirect | | 0.091| -0.325| 0.524|

|-> MBNMA | | 0.055| -0.344| 0.492|

| | | | | |

|HL_700 vs Placebo_0 | 0.378| | | |

|-> direct | | 0.443| -1.276| 2.254|

|-> indirect | | 0.826| 0.381| 1.213|

|-> MBNMA | | 0.798| 0.406| 1.165|

| | | | | |

|HL_650 vs Placebo_0 | 0.143| | | |

|-> direct | | -0.548| -2.313| 1.232|

|-> indirect | | 0.865| 0.469| 1.244|

|-> MBNMA | | 0.798| 0.406| 1.165|

| | | | | |

|HL_550 vs Placebo_0 | 0.406| | | |

|-> direct | | 0.647| -1.035| 2.379|

|-> indirect | | 0.797| 0.380| 1.185|

|-> MBNMA | | 0.796| 0.405| 1.165|

| | | | | |

|HL_500 vs Placebo_0 | 0.519| | | |

|-> direct | | 0.872| -0.341| 2.126|

|-> indirect | | 0.786| 0.348| 1.212|

|-> MBNMA | | 0.796| 0.401| 1.164|

| | | | | |

|HHL_1550 vs Placebo_0 | 0.853| | | |

|-> direct | | 0.685| -0.367| 1.705|

|-> indirect | | 0.625| -0.113| 1.390|

|-> MBNMA | | 0.703| 0.062| 1.310|

| | | | | |

|HHL_1100 vs Placebo_0 | 0.796| | | |

|-> direct | | 0.725| -0.400| 1.897|

|-> indirect | | 0.687| -0.048| 1.426|

|-> MBNMA | | 0.701| 0.062| 1.306|

| | | | | |

|HHL_1000 vs Placebo_0 | 0.513| | | |

|-> direct | | 0.372| -1.378| 2.210|

|-> indirect | | 0.739| 0.077| 1.389|

|-> MBNMA | | 0.700| 0.062| 1.306|

| | | | | |

|HH_1650 vs Placebo_0 | 0.283| | | |

|-> direct | | 1.248| 0.313| 2.210|

|-> indirect | | 0.538| 0.093| 0.984|

|-> MBNMA | | 0.670| 0.257| 1.072|

| | | | | |

|HH_1550 vs Placebo_0 | 0.378| | | |

|-> direct | | 0.218| -1.464| 1.880|

|-> indirect | | 0.697| 0.263| 1.123|

|-> MBNMA | | 0.670| 0.257| 1.071|

| | | | | |

|HH_1200 vs Placebo_0 | 0.578| | | |

|-> direct | | 0.462| -0.493| 1.454|

|-> indirect | | 0.708| 0.259| 1.152|

|-> MBNMA | | 0.669| 0.257| 1.070|

| | | | | |

|HH_900 vs Placebo_0 | 0.348| | | |

|-> direct | | 1.231| -0.414| 3.006|

|-> indirect | | 0.621| 0.195| 1.033|

|-> MBNMA | | 0.667| 0.257| 1.061|

| | | | | |


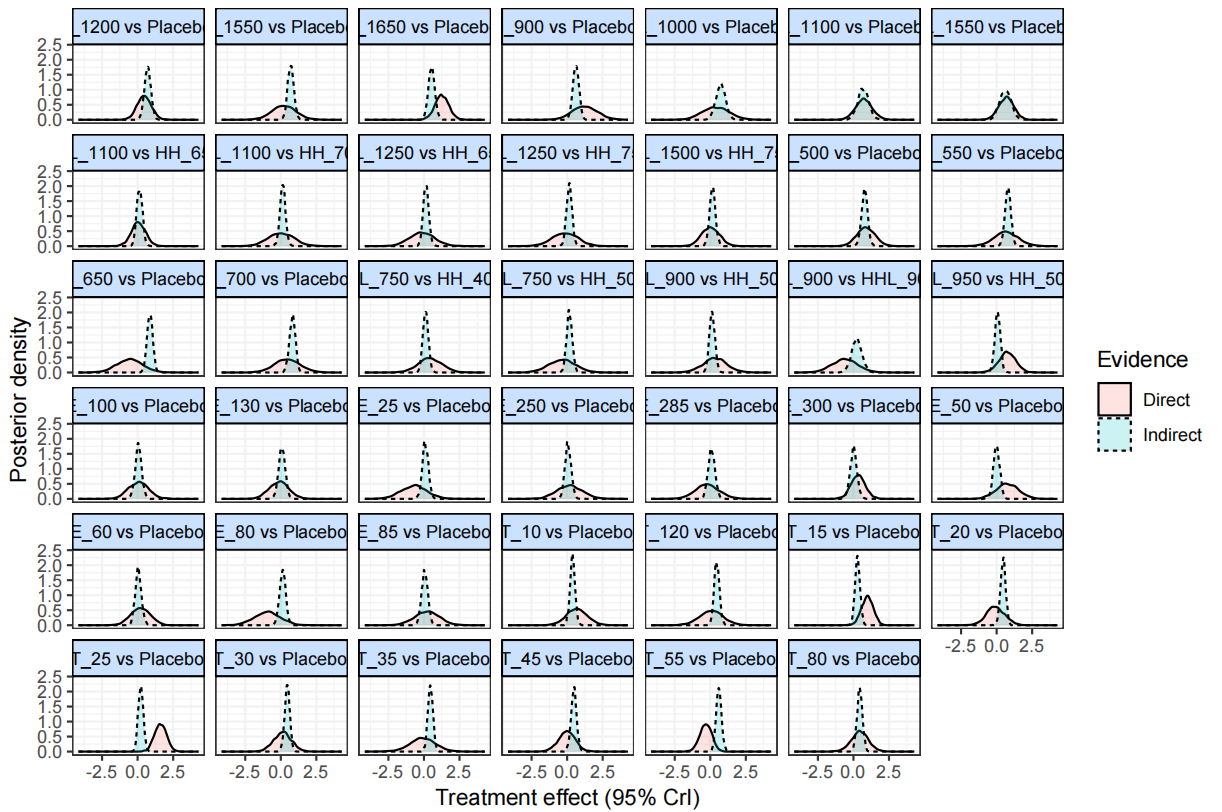


**Supplementary Figure 2.3.** Node-splitting analysis (density plot). The first value indicates the agent and the second one is the corresponding dose of that agent. HH “training high and living high”; HL “living high but living low”; IHT “interment hypoxic training”; IHE “interment hypoxic expose”;HHL “living high and training low and high”.

**Supplementary File 3: Non-linear functions and models fit comparison**

The different doses of non-pharmacological treatments were meta-analysed as independent and unrelated treatments (i.e., “split” NMA). This step is useful to determine which function fits the data better and should subsequently be used in a Model-Based Network Meta-Analysis (MBNMA) (Pedder, 2021). Figure 9.1 show the different responses (SMD) of each dose for different treatments, respectively.


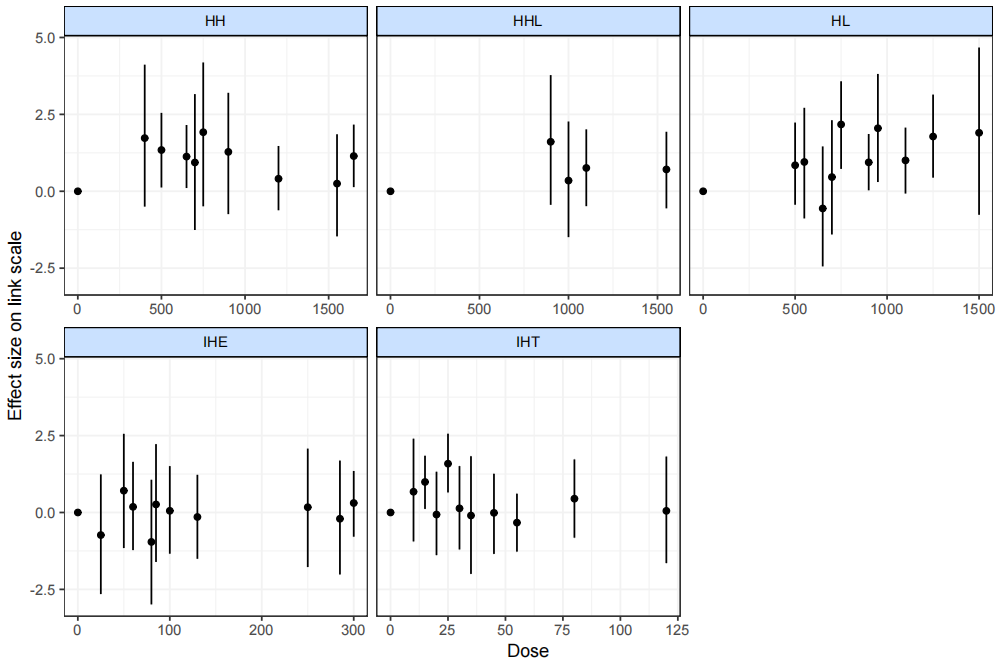


**Supplementary Figure 3.1** “Split” NMA of different altitude/hypoxic training agent.HH “training high and living high”; HL “living high but living low”; IHT “interment hypoxic training”; IHE “interment hypoxic expose”;HHL “living high and training low and high”.

Table 3.1 shows the fit indices from each of the models fitted. For our data, restricted cubic splines show the best fit and were therefore used in subsequent analyses.

**Supplementary Table 3.1 model fit comparison**

| Model | DIC | SD | Deviance | Residual deviance | Pd |
| --- | --- | --- | --- | --- | --- |
| EMAX  (common treatment effect) | 601.6 | NA | 522.984 | 337.427 | 79.0 |
| LINER  (common treatment effect) | 702.6 | NA | 624.824 | 439.266 | 78.5 |
| EXPONENTIAL  (common treatment effect) | 599.9 | NA | 521.825 | 336.267 | 78.6 |
| RESTRICTED CUBIC SPLINE  (COMMON TREATMENT EFFECTS; 3  KNOTS) | 581.8 | NA | 493.552 | 307.994 | 88.9 |
| RESTRICTED CUBIC SPLINE  (RANDOM TREATMENT EFFECTS: 3  KNOTS) | 473.7 | 0.749 (0.582,0.943) | 339.803 | 154.245 | 134.4 |
| NON-PARAMETRIC  MONOTONICALLY UP (COMMON  TREATMENT EFFECTS) | 632.2 | NA | 551.111 | 365.554 | 81.9 |

DIC = Deviance Information Criterion; SD = Between-study Standard Deviation; pD: Number of estimated parameters; NA = Not Applicable. The SD is presented as the main value and (95% Credible Intervals).

Further to model fit indices, deviance plots showing the contribution of each data point to the residual deviance are also useful to confirm the robustness of model selection (Pedder, 2021). Each data point should contribute about 1 to the posterior mean deviance, which indicates good model fit (Dias, Sutton, Ades, & Welton, 2013). The deviance plot for treatment effects (Supplementary Figure 8) confirm the robustness of our model selection (i.e., deviances < 1.5 except for 750 kilometer hours in HL,25 kilometer hours in IHT and a point of 55 kilometer hours in IHT, all below a contribution of 2.5) (Figure 3.2)


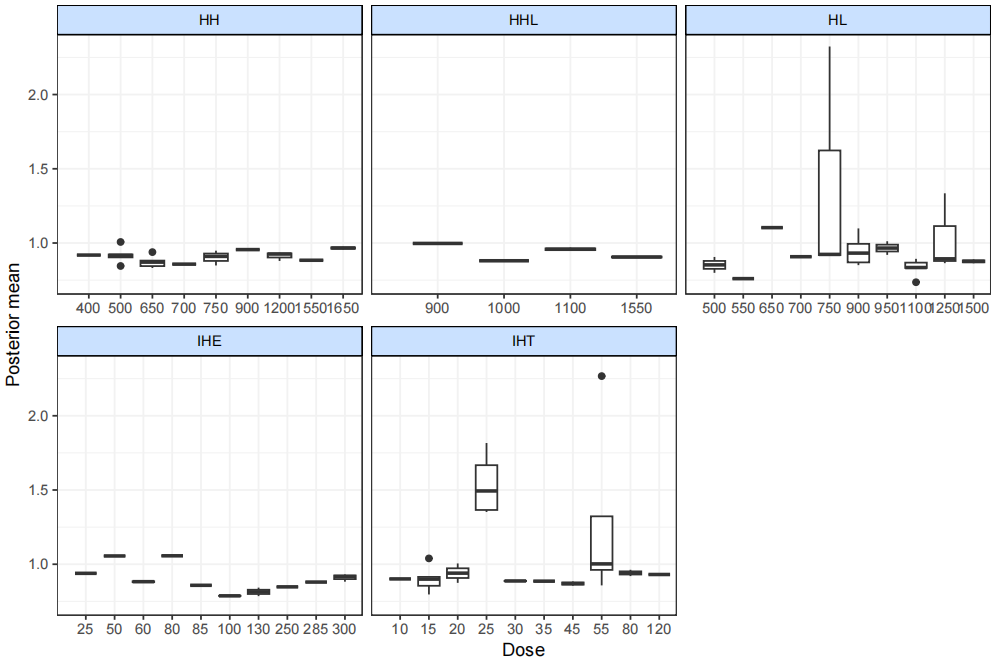


**Supplementary Figure 3.2. Deviance plots at treatment-level.**

**Supplementary file 4: Characteristics of included studies**

| Supplementary Table 4.1. Overview of selected studies | | | | | | | | | |
| --- | --- | --- | --- | --- | --- | --- | --- | --- | --- |
| study | Intervention | Sample size | Subjects | Experimental design | Hypoxic dose | | | | |
|  |  |  |  |  | Heigh(m/%) | Exposure/  intervention  days | Hypoxic  (h/d or h/session) | Total hypoxic exposure | kilometer hours |
| Benjamin  (Levine & Stray-Gundersen, 1997) | HH | f 9,m 4 | track and cross-country  running | RCT/SB | 2500m | 28 | 24 | 672 | 1680 |
|  | HL_NAT/LTA | f 9,m 4 |  |  | 1250m/2500m | 28 | 4/20 | 672 | 1534 |
|  | NA | f 9,m 4 |  |  | sea level | 28 | - | - | - |
| Eileen Y. Robertson  (Robertson, Saunders, Pyne, Gore, & Anson, 2010) | HHL | 8 | middle-distance  and distance runners | RCT/UB | 2200m/3000m | 21（4 session/week） | 1.25/14 | 309 | 915 |
|  | HL_SIM/TSL | 8 |  |  | 3000m | 21 | 14 | 294 | 882 |
| Ferran  (F. A. Rodríguez et al., 2015) | HH | f 4,m 8 | swimming | UCT | 2320m | 21 | 24 | 504 | 1169 |
|  | HH | f 8,m 7 |  |  |  | 28 | 24 | 672 | 1559 |
|  | HHL | f10，m6 |  |  | 690m/2320m | 28 | 24 | 672 | 1559 |
|  | NA | f 8,m 3 |  |  | sea level | 28 | - | - | - |
| Kazunobu Okazaki  (Okazaki, Stray-Gundersen, Chapman, & Levine, 2019) | HH | f 8，m 18 | distance runners | RCT/UB | 2500m | 28 | 24 | 672 | 1680 |
|  | NA | f 4，m 9 |  |  | sea level | 28 | - | - | - |
| Thomas Christian Bonne  (Bonne et al., 2014) | HH | f 5，m 5 | swimming | UCT | 2612m | 28 | 24 | 672 | 1755 |
|  | NA | f 6，m 4 |  |  | sea level | 28 | - | - | - |
| Zhijun Meng  (Meng et al., 2021) | HH | m 18 | rower | UCT | 2280m | 56 | 24 | 1334 | 3041 |
|  | NA | m 15 |  |  | sea level | 56 | - | - | - |
| Christoph Siebenmann  (C. Siebenmann et al., 2012) | HH | m 13 | rowr and runer | UCT | 1740m | 28 | 24 | 672 | 1169 |
|  | HH | m 8 |  |  | 1300m | 28 | 24 | 672 | 873.6 |
|  | NA | m 8 |  |  | sea level | 28 | - | - | - |
| 1. Burtscher   (Burtscher, Nachbauer, Baumgartl, & Philadelphy, 1996) | HH | m 10 | runer | UCT | 2315m | 12 | 24 | 288 | 667 |
|  | NA | m 12 |  |  | sea level | 12 | - | - | - |
| Amelia J. Carr  (Carr et al., 2019) | HH | f 3，m 4 | race walkers | UCT | 1380m | 14 | 24 | 336 | 464 |
|  | HL_SIM/TSL | f 2，m 5 |  |  | 3000m | 14 | 14 | 196 | 588 |
|  | NA | f 2，m 6 |  |  | sea level | 14 | - | - | - |
| Hun-Young Park  (Park, Park, & Lim, 2019) | HL_SIM/TSL | m 12 | middle- and long-distance runners | RCT/UB | 3000m | 21 | 12 | 252 | 756 |
|  | NA | m 12 |  |  | sea level | 21 | - | - | - |
| Czuba M  (Czuba, Fidos-Czuba, Płoszczyca, Zając, & Langfort, 2018) | HL_SIM/TSL | 10 | competitive off-road cyclists | RCT/UB | 2100m | 21 | 11.5 | 241.5 | 507.15 |
|  | IHT | 10 |  |  | 2100m | 21(3 session/week) | 35min | 5.25 | 11 |
|  | NA | 10 |  |  | sea level | 21 | - | - | - |
| Paul Robach  (P Robach et al., 2012) | HL_NAT/LTA | f 1,m 9 | cyclists and triathletes | RCT/DB | 1100m/3000m | 28 | 10/14 | 672 | 1484 |
|  | HH | m 6 |  |  | 1100m | 28 | 24 | 672 | 739.2 |
| Clare E. Humberstone-Gough  (Humberstone-Gough et al., 2013) | HL_SIM/TSL | f 2,m 5 | triathletes | RCT/SB | 3000m | 17 | 14 | 238 | 714 |
|  | IHE | f 2,m 5 |  |  | 4750m | 17(1 session/day) | 36min | 10.2 | 48.45 |
|  | NA | f 3,m 6 |  |  | sea level | 17 | - | - | - |
| 1. J.GORE   (Science, 2001) | HL_SIM/TSL | m=6 | triathletes/  cross country skiers/cyclists | UCT | 3000m | 23 | 9.5 | 218.5 | 656 |
|  | NA | m=7 |  |  | sea level | 23 | - | - | - |
| EILEEN Y. ROBERTSON  (E. Y. Robertson et al., 2010) | HL_SIM/TSL | f 2,m 6 | middle distance and distance runners | RCT/UB | 3000m | 21 | 14 | 294 | 882 |
|  | NA | f 3,m 5 |  |  | sea level | 21 | - | - | - |
| Philo U. Saunders  (P. U. Saunders et al., 2010) | HL_SIM/TSL | f 3,m 3 | race walkers | RCT/SB | 3000m | 21 | 14 | 294 | 882 |
|  | NA | f 3,m 2 |  |  | sea level | 21 | - | - | - |
|  | Placebo | f 3,m 3 |  |  | sea level | 21 | - | - | - |
| 1. U. Saunders   (P. Saunders, Telford, Pyne, Hahn, & Gore, 2009) | HL_SIM/TSL | 9 | middle distance runners | RCT/UB | 2860 | 56(5 sessions/week) | 9 | 360 | 1092 |
|  | NA | 9 |  |  | sea level | 56 | - | - | - |
| Jonas J. Saugy  (J. Saugy et al., 2016) | HL_SIM/TLA | m 16 | triathletes | UCT | 1150m/2250m | 18 | 11.3/12.7 | 432 | 749 |
|  | HL_NAT/LTA | m 16 |  |  | 1150m/2250m | 18 | 6.9/17.1 | 432 | 842.5 |
| Laurent Schmitt  (Schmitt, Willis, Fardel, Coulmy, & Millet, 2018) | HL_SIM/TLA | m 5,f 13 | Nordic-skiers | RCT/UB | 1150m/2700m | 15 | 10/14 | 360 | 740 |
|  | HH | m 5 |  |  | 1150m | 15 | 24 | 360 | 414 |
| Amelia J. Carr  (Carr, Saunders, Vallance, Garvican-Lewis, & Gore, 2015) | HH | m 3,f 6 | race walkers | UCT | 1380m | 21 | 24 | 504 | 696 |
|  | HL_SIM/TLA | m 3,f 6 |  |  | 1380m/3000m | 21 | 15/9 | 504 | 1002 |
|  | NA | m 5,f 5 |  |  | sea level | 21 | - | - | - |
| Dariusz Sitkowski  (Sitkowski et al., 2019) | HL_SIM/TLA | f 8 | endurance event track and road | RCT/SB | 1100m/2200m | 21 | 11/13 | 504 | 785 |
|  | Placebo(HH) | f 7 |  |  | 1100m | 21 | 24 | 504 | 554 |
|  | HH | f 7 |  |  | 900m | 21 | 24 | 504 | 453.6 |
| Christoph Siebenmann  (C Siebenmann et al., 2012) | HL_SIM/TLA | f 1,m 9 | endurance | RCT/DB | 1200m/3000m | 28 | 10/14 | 672 | 1484 |
|  | HH | m 6 |  |  | 1200m | 28 | 24 | 672 | 806 |
| Mitsuo Neya  (Neya, Enoki, Ohiwa, Kawahara, & Gore, 2012) | HL_SIM/TLA | m 7 | long- and middle-distance runners | UCT | 1300m/3000m | 22 | 14/10 | 528 | 1056 |
|  | HH | m 7 |  |  | 1300m | 22 | 24 | 528 | 686 |
| Jonas J. Saugy  (J. J. Saugy et al., 2014) | HL_SIM/TLA | m 12 | triathletes | UCT | 1150m/2250m | 18 | 12/12 | 432 | 734 |
|  | HL_NAT/LTA | m 12 |  |  | 1150m/2250m | 18 | 7/17 | 432 | 834 |
| 1. Schmitt   (Schmitt et al., 2008) | HL_SIM/TLA | f 3,m 3 | cross-country skiers | RCT/UB | 1200m/3000m | 18 | 13/11 | 432 | 875 |
|  | HH | f 3,m 2 |  |  | 1200m | 18 | 24 | 432 | 518 |
| Laurent Schmitt  (Schmitt et al., 2006) | HL_SIM/TLA | f 1,m 19 | cross-country skiers, swimmers, runners | UCT | 1200m/3000m | 18 | 11/13 | 432 | 940 |
|  | HH | f 1,m 19 |  |  | 1200m | 18 | 24 | 432 | 518 |
| Julien V. Brugniaux  (Brugniaux et al., 2006) | HL_SIM/TLA | m 5 | long- and middle-distance runners | RCT/UB | 1200m/2800m | 18 | 10/14 | 432 | 922 |
|  | HH | m 6 |  |  | 1200m | 18 | 24 | 432 | 518 |
| Paul Robach  (Paul Robach et al., 2018) | HL_NAT/LTA | f 4,m 7 | cross-country skiers | UCT | 1035m/2207m | 26 | 7/17 | 624 | 1164 |
|  | HH | f 1,m 8 |  |  | 1035m | 26 | 24 | 624 | 646 |
| 1. Dehnert   (Dehnert et al., 2002) | HL_NAT/LTA | f 3,m 8 | triathletes | RCT/UB | 800m/2000m | 14 | 11/13 | 336 | 487 |
|  | HH | f 3,m 7 |  |  | 800m | 14 | 24 | 336 | 269 |
| Milosz Czuba  (Czuba et al., 2014) | HHL | m 7 | triathletes | RCT/UB | 3000m/1000m/2015m | 21 | 24 | 504(19/30/455) | 1008 |
|  | NA | m 7 |  |  | sea level | 21 | - | - | - |
| Hun-Young Park  (Park, Shin, & Lim, 2018) | IHT | f 5；m 5 | swimming | RCT/UB | 3000m | 42  (3 sessions/week) | 1.5 | 27 | 81 |
|  | NA | f 5；m 5 |  |  | sea level | 42 | - | - | - |
| PREETIWAT WONNABUSSAPAWICH  (Wonnabussapawich et al., 2017) | HHL | m 20 | Soccer | RCT/UB | 825m/3000m | 56  (3 sessions/week) | 0.25 | 1134 | 1109 |
|  | NA | m 20 |  |  | sea level | 56 | - | - | - |
| Miłosz Czuba  (Czuba et al., 2013) | IHT | m 6 | basketball players | RCT/UB | 2500m | 21  (6 sessions/week) | 16min | 4.86 | 12.15 |
|  | NA | m 6 |  |  | sea level | 21 | - | - | - |
| Won-Sang Jung  (Jung, Kim, & Park, 2020) | IHT | m 10 | middle- and long-distance runners | UCT | 3000m | 42  (3 sessions/week) | 1 | 18 | 54 |
|  | NA | m 10 |  |  | sea level | 42 | - | - | - |
| Hun-Young Park  (Park, Jung, Kim, Kim, & Lim, 2022) | IHT | f 10 | runners | UCT | 3000m | 42  (3 sessions/week) | 1 | 18 | 54 |
|  | NA | f 10 |  |  | sea level | 42 | - | - | - |
| Gre´goire Millet  (Millet et al., 2014) | IHT | m 9 | cyclists | RCT/UB | 3000m | 21  (2 sessions/week) | - | 19.1 | 57.3 |
|  | NA | m 9 |  |  | sea level | 21 | - | - | - |
| Milosz Czuba  (Czuba et al., 2011) | IHT | m 10 | elite cyclists | RCT/UB | 2650m | 21  (3 sessions/week) | 35min | 5.25 | 13.9 |
|  | NA | m 10 |  |  | sea level | 21 | - | - | - |
| BEN A. HOLLISS  (Holliss, Burden, Jones, & Pedlar, 2014) | IHT | m 5 | middle- and long-distance runners | RCT/SB | 2150m | 56  (2 sessions/week) | 40min | 10.7 | 22.9 |
|  | NA | m 7 |  |  | sea level | 56 | - | - | - |
| Miłosz Czuba  (Czuba et al., 2017) | IHT | m 8 | swimmers | RCT/UB | 2500m | 28  (2 sessions/week) | 50min | 6.7 | 16.7 |
|  | NA | m 8 |  |  | sea level | 28 | - | - | - |
| Ingrid J.M. Hendriksen  (Hendriksen & Meeuwsen, 2003) | IHT | m 12 | triathletes | UCT | 2500m | 10(1 sessions/day) | 105min | 17.5 | 43.75 |
|  | NA | m 12 |  |  | sea level | 10 | - | - | - |
| DJ Ramos-Campo  (Ramos-Campo et al., 2015) | IHT | m 9 | triathletes | RCT/UB | 3875m（14.5%-15%） | 49  (2 sessions/week) | 1 | 14 | 54.25 |
|  | NA | m 9 |  |  | sea level | 49 | - | - | - |
| 1. J. TRUIJENS   (Truijens, Toussaint, Dow, & Levine, 2003) | IHT | f 3,m 5 | swimmers | RCT/DB | 3530m（15.3%） | 35(3 sessions/week) | 22.75min | 5.68 | 20 |
|  | NA | f 3,m 5 |  |  | sea level | 35 | - | - | - |
| Miłosz Czuba  (Czuba et al., 2019) | IHT | m 7 | biathletes | RCT/SB | 2000m | 21(3 sessions/week) | 45min | 6.75 | 13.5 |
|  | NA | m 7 |  |  | sea level | 21 | - | - | - |
| Sung-Woo Kim  (Kim, Jung, Kim, Nam, & Park, 2021) | IHT | m 10 | swimmers | UCT | 3000m | 42(3 sessions/week) | 1.5 | 27 | 81 |
|  | NA | m 10 |  |  | sea level | 42 | - | - | - |
| Aneta Teległów  (Teległów et al., 2022) | IHT | m 7 | rowing | RCT/UB | 2400 | 21(3 sessions/week) | 1 | 9 | 21.6 |
|  | NA | m 7 |  |  | sea level | 21 | - | - | - |
| Damon Arezzolo  (Arezzolo, Coffey, Byrne, & Doering, 2020) | IHT | m 9 | cyclists | RCT/SB | 3625(14.7%) | 28(2 sessions/week) | 1 | 8 | 29 |
|  | NA | m 9 |  |  | sea level | 28 | - | - | - |
| Elodie Ponsot  (Ponsot et al., 2006) | IHT | m 8 | distance runners | RCT/UB | 3000m | 42(2 sessions/week) | 40min | 8 | 24 |
|  | NA | m 7 |  |  | sea level | 42 | - | - | - |
| Ste´phane P. Dufour | IHT | m 9 | distance runners | RCT/UB | 3000m | 42(2 sessions/week) | 40min | 8 | 24 |
|  | NA | m 9 |  |  | sea level | 42 | - | - | - |
| James Peter Morton  (Morton & Cable, 2005) | IHT | m 8 | team sports players | UCT | 2750m | 56(3 sessions/week) | 30min | 12 | 33 |
|  | NA | m 8 |  |  | sea level | 56 | - | - | - |
| Tadeusz Ambrozy  (Ambroży et al., 2020) | IHT | m 15 | boxer | RCT/UB | 4000m | 42(5 sessions/week) | 1 | 30 | 120 |
|  | NA | m 15 |  |  | sea level | 45 | - | - | - |
| Colleen G. Julian  (Julian et al., 2004) | IHE | m 7 | distance runners | RCT/DB | 6875m（11%） | 56(3 sessions/week) | 0.5 | 12 | 82.5 |
|  | NA | f 1,m 7 |  |  | sea level | 56 | - | - | - |
| M.Burtscher  (Burtscher, Gatterer, Faulhaber, Gerstgrasser, & Schenk, 2010) | IHE | f 1,m 5 | middle-distance runners | RCT/DB | 4350m | 35(3 sessions/week) | 2 | 30 | 130.5 |
|  | NA | f 2,m 3 |  |  | sea level | 35 | - | - | - |
| Ferran A. Rodríguez  (F. Rodríguez et al., 2007) | IHE | f 6,m 5 | runners and swimmers | RCT/DB | 4750m | 28(5 sessions/week) | 3 | 60 | 285 |
|  | NA | f 6,m 6 |  |  | sea level | 26 | - | - | - |
| Keisho Katayama  (Katayama et al., 2004) | IHE | m 8 | endurance runners | RCT/SB | 5400m | 14(1 sessions/day) | 3 | 42 | 226.8 |
|  | NA | m 7 |  |  | sea level | 14 | - | - | - |
| Darrell L. Bonetti  (Bonetti, Hopkins, & Kilding, 2006) | IHE | m 5 | kayak paddlers | C | 6875m（11%） | 21(5 sessions/week) | 1 | 15 | 103.1 |
|  | NA | m 5 |  |  | sea level | 21 | - | - | - |
| Martin J. Truijens  (Truijens et al., 2008) | IHE | f 5,m 6 | swimming and running | RCT/DB | 4750m | 28(5 sessions/week) | 3 | 60 | 285 |
|  | NA | f 6,m 6 |  |  | sea level | 28 | - | - | - |
| ANDREW E. KILDING  (Kilding, Dobson, & Ikeda, 2016) | IHE | m 7 | basketball players | RCT/SB | 3000m | 15(1 sessions/day) | 0.5 | 7.5 | 22.5 |
|  | NA | m 7 |  |  | sea level | 15 | - | - | - |
| VAHID TADIBI  (Tadibi, Dehnert, Menold, & Bärtsch, 2007) | IHE | m 10 | endurance athletes | RCT/DB | 5500m | 15(1 sessions/day) | 1 | 15 | 82.5 |
|  | NA | m 10 |  |  | sea level | 15 | - | - | - |
| KEISHO KATAYAMA  (Katayama, Matsuo, Ishida, Mori, & Miyamura, 2003) | IHE | m 6 | endurance runners | UCT | 4500m | 21(3 sessions/week) | 1.5 | 13.5 | 60.75 |
|  | NA | m 6 |  |  | sea level | 21 | - | - | - |

The *Heigh (m /%)* refers to the altitude where the subject is actually exposed, with "/", to represent training and life.The *Exposure / intervention days* is the days of participation in the activities and the frequency of the training sessions.The *Hypoxic (h / d or h / session)* is the duration of hypoxia exposure, daily or per session.*The total hypoxic exposure* is the total hypoxia exposure duration of the subjects throughout the experimental session.HH “training high and living high”; HL_NAT/TLA “living at natural altitude but living at low altitude”;HL_SIM/TLA “living at artificial altitude but living at low altitude”; HL_SIM/TSL “living at artificial altitude but living at sea level”；IHT “interment hypoxic training”; IHE “interment hypoxic expose”;HHL “living high and training low and high”;RCT / DB "randomized double blind controlled experiment"; RCT / SB "randomized single blind control experiment"; UCT "non-randomized controlled experiment"; C "Cross experiment".

**Supplementary File 5: Study-level risk of bias analysis**


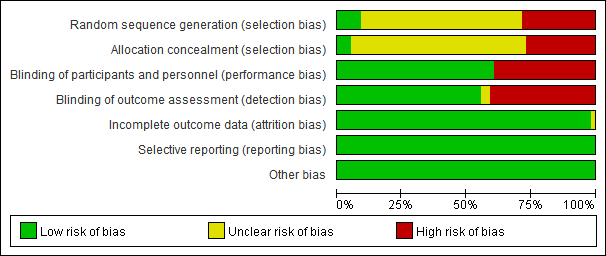


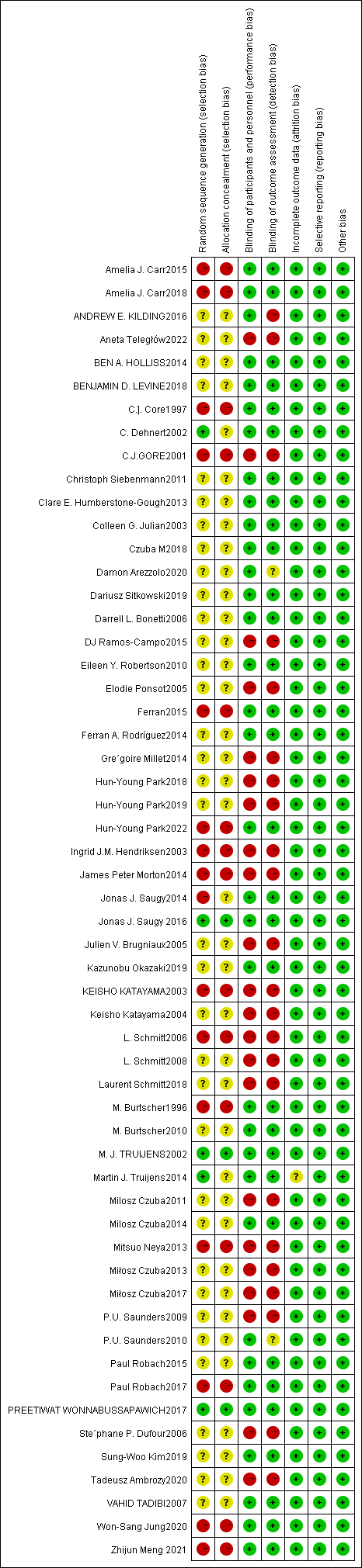


Ambroży, T., Maciejczyk, M., Klimek, A., Wiecha, S., Stanula, A., Snopkowski, P., . . . Cynarski, W. (2020). The Effects of Intermittent Hypoxic Training on Anaerobic and Aerobic Power in Boxers. *International Journal of Environmental Research and Public Health, 17*(24). doi:10.3390/ijerph17249361

Arezzolo, D., Coffey, V., Byrne, N., & Doering, T. (2020). Effects of Eight Interval Training Sessions in Hypoxia on Anaerobic, Aerobic, and High Intensity Work Capacity in Endurance Cyclists. *High Altitude Medicine & Biology, 21*(4), 370-377. doi:10.1089/ham.2020.0066

Bonetti, D., Hopkins, W., & Kilding, A. (2006). High-intensity kayak performance after adaptation to intermittent hypoxia. *International Journal of Sports Physiology and Performance, 1*(3), 246-260. doi:10.1123/ijspp.1.3.246

Bonne, T., Lundby, C., Jørgensen, S., Johansen, L., Mrgan, M., Bech, S., . . . Nordsborg, N. (2014). "Live High-Train High" increases hemoglobin mass in Olympic swimmers. *European Journal of Applied Physiology, 114*(7), 1439-1449. doi:10.1007/s00421-014-2863-4

Brugniaux, J. V., Schmitt, L., Robach, P., Nicolet, G., Fouillot, J. P., Moutereau, S., . . . Chorvot, M. C. (2006). Eighteen days of "living high, training low" stimulate erythropoiesis and enhance aerobic performance in elite middle-distance runners. *Journal of Applied Physiology, 100*(1), 203-211.

Burtscher, M., Gatterer, H., Faulhaber, M., Gerstgrasser, W., & Schenk, K. (2010). Effects of intermittent hypoxia on running economy. *International Journal of Sports Medicine, 31*(9), 644-650. doi:10.1055/s-0030-1255067

Burtscher, M., Nachbauer, W., Baumgartl, P., & Philadelphy, M. (1996). Benefits of training at moderate altitude versus sea level training in amateur runners. *European journal of applied physiology and occupational physiology, 74*(6), 558-563. doi:10.1007/bf02376773

Carr, A., Garvican-Lewis, L., Vallance, B., Drake, A., Saunders, P., Humberstone, C., & Gore, C. (2019). Training to Compete at Altitude:Natural Altitude or Simulated Live High:Train Low? *International Journal of Sports Physiology and Performance, 14*(4), 509-517. doi:10.1123/ijspp.2018-0099

Carr, A., Saunders, P., Vallance, B., Garvican-Lewis, L., & Gore, C. (2015). Increased Hypoxic Dose After Training at Low Altitude with 9h Per Night at 3000m Normobaric Hypoxia. *Journal of sports science & medicine, 14*(4), 776-782.

Czuba, M., Bril, G., Płoszczyca, K., Piotrowicz, Z., Chalimoniuk, M., Roczniok, R., . . . Langfort, J. (2019). Intermittent Hypoxic Training at Lactate Threshold Intensity Improves Aiming Performance in Well-Trained Biathletes with Little Change of Cardiovascular Variables. *BioMed research international, 2019*, 1287506. doi:10.1155/2019/1287506

Czuba, M., Fidos-Czuba, O., Płoszczyca, K., Zając, A., & Langfort, J. (2018). Comparison of the effect of intermittent hypoxic training vs. the live high, train low strategy on aerobic capacity and sports performance in cyclists in normoxia. *Biology of Sport, 35*(1), 39-48. doi:10.5114/biolsport.2018.70750

Czuba, M., Maszczyk, A., Gerasimuk, D., Roczniok, R., Fidos-Czuba, O., Zając, A., . . . Langfort, J. (2014). The effects of hypobaric hypoxia on erythropoiesis, maximal oxygen uptake and energy cost of exercise under normoxia in elite biathletes. *Journal of sports science & medicine, 13*(4), 912-920.

Czuba, M., Waskiewicz, Z., Zajac, A., Poprzecki, S., Cholewa, J., & Roczniok, R. (2011). The effects of intermittent hypoxic training on aerobic capacity and endurance performance in cyclists. *Journal of sports science & medicine, 10*(1), 175-183.

Czuba, M., Wilk, R., Karpiński, J., Chalimoniuk, M., Zajac, A., & Langfort, J. (2017). Intermittent hypoxic training improves anaerobic performance in competitive swimmers when implemented into a direct competition mesocycle. *PLoS One, 12*(8), e0180380. doi:10.1371/journal.pone.0180380

Czuba, M., Zając, A., Maszczyk, A., Roczniok, R., Poprzęcki, S., Garbaciak, W., & Zając, T. (2013). The effects of high intensity interval training in normobaric hypoxia on aerobic capacity in basketball players. *Journal of Human Kinetics, 39*, 103-114. doi:10.2478/hukin-2013-0073

Dehnert, C., Hütler, M., Liu, Y., Menold, E., Netzer, C., Schick, R., . . . Steinacker, J. (2002). Erythropoiesis and performance after two weeks of living high and training low in well trained triathletes. *International Journal of Sports Medicine, 23*(8), 561-566. doi:10.1055/s-2002-35533

Hendriksen, I., & Meeuwsen, T. (2003). The effect of intermittent training in hypobaric hypoxia on sea-level exercise: a cross-over study in humans. *European Journal of Applied Physiology, 88*, 396-403. doi:10.1007/s00421-002-0708-z

Holliss, B., Burden, R., Jones, A., & Pedlar, C. (2014). Eight weeks of intermittent hypoxic training improves submaximal physiological variables in highly trained runners. *Journal of Strength and Conditioning Research, 28*(8), 2195-2203. doi:10.1519/jsc.0000000000000406

Humberstone-Gough, C., Saunders, P., Bonetti, D., Stephens, S., Bullock, N., Anson, J., & Gore, C. (2013). Comparison of live high: train low altitude and intermittent hypoxic exposure. *Journal of sports science & medicine, 12*(3), 394-401.

Julian, C., Gore, C., Wilber, R., Daniels, J., Fredericson, M., Stray-Gundersen, J., . . . Levine, B. (2004). Intermittent normobaric hypoxia does not alter performance or erythropoietic markers in highly trained distance runners. *Journal of applied physiology (Bethesda, Md. : 1985), 96*(5), 1800-1807. doi:10.1152/japplphysiol.00969.2003

Jung, W., Kim, S., & Park, H. (2020). Interval Hypoxic Training Enhances Athletic Performance and Does Not Adversely Affect Immune Function in Middle- and Long-Distance Runners. *International Journal of Environmental Research and Public Health, 17*(6). doi:10.3390/ijerph17061934

Katayama, K., Matsuo, H., Ishida, K., Mori, S., & Miyamura, M. (2003). Intermittent hypoxia improves endurance performance and submaximal exercise efficiency. *High Altitude Medicine & Biology, 4*(3), 291-304. doi:10.1089/152702903769192250

Katayama, K., Sato, K., Matsuo, H., Ishida, K., Iwasaki, K., & Miyamura, M. (2004). Effect of intermittent hypoxia on oxygen uptake during submaximal exercise in endurance athletes. *European Journal of Applied Physiology, 92*, 75-83. doi:10.1007/s00421-004-1054-0

Kilding, A., Dobson, B., & Ikeda, E. (2016). Effects of Acutely Intermittent Hypoxic Exposure on Running Economy and Physical Performance in Basketball Players. *Journal of Strength and Conditioning Research, 30*(7), 2033-2042. doi:10.1519/jsc.0000000000001301

Kim, S., Jung, W., Kim, J., Nam, S., & Park, H. (2021). Aerobic Continuous and Interval Training under Hypoxia Enhances Endurance Exercise Performance with Hemodynamic and Autonomic Nervous System Function in Amateur Male Swimmers. *International Journal of Environmental Research and Public Health, 18*(8). doi:10.3390/ijerph18083944

Levine, B., & Stray-Gundersen, J. (1997). "Living high-training low": effect of moderate-altitude acclimatization with low-altitude training on performance. *Journal of applied physiology (Bethesda, Md. : 1985), 83*(1), 102-112. doi:10.1152/jappl.1997.83.1.102

Meng, Z., Gao, H., Li, T., Ge, P., Xu, Y., & Gao, B. (2021). Effects of Eight Weeks Altitude Training on the Aerobic Capacity and Microcirculation Function in Trained Rowers. *High Altitude Medicine & Biology, 22*(1), 24-31. doi:10.1089/ham.2020.0059

Millet, G., Bentley, D., Roels, B., Mc Naughton, L., Mercier, J., & Cameron-Smith, D. (2014). Effects of intermittent training on anaerobic performance and MCT transporters in athletes. *PLoS One, 9*(5), e95092. doi:10.1371/journal.pone.0095092

Morton, J., & Cable, N. (2005). Effects of intermittent hypoxic training on aerobic and anaerobic performance. *Ergonomics, 48*, 1535-1546. doi:10.1080/00140130500100959

Neya, M., Enoki, T., Ohiwa, N., Kawahara, T., & Gore, C. J. (2012). Increased Hemoglobin Mass and VO2max With 10 h Nightly Simulated Altitude at 3000 m. *International Journal of Sports Physiology & Performance, 8*(4), 366-372.

Okazaki, K., Stray-Gundersen, J., Chapman, R. F., & Levine, B. D. (2019). Iron insufficiency diminishes the erythropoietic response to moderate altitude exposure. *Journal of Applied Physiology, 127*(6), 1569-1578.

Park, H., Jung, W., Kim, S., Kim, J., & Lim, K. (2022). Effects of Interval Training Under Hypoxia on Hematological Parameters, Hemodynamic Function, and Endurance Exercise Performance in Amateur Female Runners in Korea. *Frontiers in Physiology, 13*, 919008. doi:10.3389/fphys.2022.919008

Park, H., Park, W., & Lim, K. (2019). Living High-Training Low for 21 Days Enhances Exercise Economy, Hemodynamic Function, and Exercise Performance of Competitive Runners. *Journal of sports science & medicine, 18*(3), 427-437.

Park, H., Shin, C., & Lim, K. (2018). Intermittent hypoxic training for 6 weeks in 3000 m hypobaric hypoxia conditions enhances exercise economy and aerobic exercise performance in moderately trained swimmers. *Biology of Sport, 35*(1), 49-56. doi:10.5114/biolsport.2018.70751

Ponsot, E., Dufour, S., Zoll, J., Doutrelau, S., N'Guessan, B., Geny, B., . . . Richard, R. (2006). Exercise training in normobaric hypoxia in endurance runners. II. Improvement of mitochondrial properties in skeletal muscle. *Journal of applied physiology (Bethesda, Md. : 1985), 100*(4), 1249-1257. doi:10.1152/japplphysiol.00361.2005

Ramos-Campo, D., Martínez-Sánchez, F., Esteban-García, P., Rubio-Arias, J., Clemente-Suarez, V., & Jiménez-Díaz, J. (2015). The effects of intermittent hypoxia training on hematological and aerobic performance in triathletes. *Acta Physiologica Hungarica, 102*(4), 409-418. doi:10.1556/036.102.2015.4.8

Robach, P., Hansen, J., Pichon, A., Meinild Lundby, A. K., Dandanell, S., Slettal?Kken Falch, G., . . . Keiser, S. (2018). Hypobaric live high‐train low does not improve aerobic performance more than live low‐train low in cross‐country skiers. *Scandinavian Journal of Medicine & ence in Sports*.

Robach, P., Siebenmann, C., Jacobs, R., Rasmussen, P., Nordsborg, N., Pesta, D., . . . Lundby, C. (2012). The role of haemoglobin mass on VO(2)max following normobaric 'live high-train low' in endurance-trained athletes. *British Journal of Sports Medicine, 46*(11), 822-827. doi:10.1136/bjsports-2012-091078

Robertson, E. Y., Saunders, P. U., Pyne, D. B., Aughey, R. J., Anson, J. M., & Gore, C. J. (2010). Reproducibility of performance changes to simulated live high/train low altitude. *Medicine & Science in Sports & Exercise, 42*(2), 394-401.

Robertson, E. Y., Saunders, P. U., Pyne, D. B., Gore, C. J., & Anson, J. M. (2010). Effectiveness of intermittent training in hypoxia combined with live high/train low. *European Journal of Applied Physiology, 110*(2), 379-387.

Rodríguez, F., Truijens, M., Townsend, N., Stray-Gundersen, J., Gore, C., & Levine, B. (2007). Performance of runners and swimmers after four weeks of intermittent hypobaric hypoxic exposure plus sea level training. *Journal of applied physiology (Bethesda, Md. : 1985), 103*(5), 1523-1535. doi:10.1152/japplphysiol.01320.2006

Rodríguez, F. A., Iglesias, X., Feriche, B., Calderón-Soto, C., Chaverri, D., Wachsmuth, N. B., . . . Levine, B. D. (2015). Altitude training in elite swimmers for sea level performance (altitude project). *Med Sci Sports Exerc, 47*(9), 1965-1978.

Saugy, J., Schmitt, L., Hauser, A., Constantin, G., Cejuela, R., Faiss, R., . . . Millet, G. (2016). Same Performance Changes after Live High-Train Low in Normobaric vs. Hypobaric Hypoxia. *Frontiers in Physiology, 7*, 138. doi:10.3389/fphys.2016.00138

Saugy, J. J., Laurent, S., Roberto, C., Raphael, F., Anna, H., Wehrlin, J. P., . . . P., M. G. (2014). Comparison of "Live High-Train Low" in Normobaric versus Hypobaric Hypoxia. *PLoS One, 9*(12), e114418.

Saunders, P., Telford, R., Pyne, D., Hahn, A., & Gore, C. (2009). Improved running economy and increased hemoglobin mass in elite runners after extended moderate altitude exposure. *Journal of Science and Medicine in Sport, 12*(1), 67-72. doi:10.1016/j.jsams.2007.08.014

Saunders, P. U., Ahlgrim, C., Vallance, B., Green, D. J., Robertson, E. Y., Clark, S. A., . . . Gore, C. J. (2010). An attempt to quantify the placebo effect from a three-week simulated altitude training camp in elite race walkers. *Int J Sports Physiol Perform, 5*(4), 521-534.

Schmitt, L., Fouillot, J., Millet, G., Robach, P., Nicolet, G., Brugniaux, J., & Richalet, J. (2008). Altitude, heart rate variability and aerobic capacities. *International Journal of Sports Medicine, 29*(4), 300-306. doi:10.1055/s-2007-965355

Schmitt, L., Millet, G., Robach, P., Nicolet, G., Brugniaux, J., Fouillot, J., & Richalet, J. (2006). Influence of "living high-training low" on aerobic performance and economy of work in elite athletes. *European Journal of Applied Physiology, 97*(5), 627-636. doi:10.1007/s00421-006-0228-3

Schmitt, L., Willis, S., Fardel, A., Coulmy, N., & Millet, G. (2018). Live high-train low guided by daily heart rate variability in elite Nordic-skiers. *European Journal of Applied Physiology, 118*(2), 419-428. doi:10.1007/s00421-017-3784-9

Science, S. (2001). Live high : train low increases muscle buffer capacity and submaximal cycling ef ® ciency.

Siebenmann, C., Robach, P., Jacobs, R., Rasmussen, P., Nordsborg, N., Diaz, V., . . . Lundby, C. (2012). "Live high-train low" using normobaric hypoxia: a double-blinded, placebo-controlled study. *Journal of applied physiology (Bethesda, Md. : 1985), 112*(1), 106-117. doi:10.1152/japplphysiol.00388.2011

Siebenmann, C., Robach, P., Jacobs, R. A., Rasmussen, P., Nordsborg, N., Diaz, V., . . . Lundby, C. (2012). "Live high-train low" using normobaric hypoxia: a double-blinded, placebo-controlled study. *Journal of Applied Physiology, 112*(1), 106-117.

Sitkowski, D., Szygula, Z., Surała, O., Orysiak, J., Zdanowicz, R., Pokrywka, A., . . . Malczewska-Lenczowska, J. (2019). Hematological status and endurance performance predictors after low altitude training supported by normobaric hypoxia: a double-blind, placebo controlled study. *Biology of Sport, 36*(4), 341-349. doi:10.5114/biolsport.2019.88760

Tadibi, V., Dehnert, C., Menold, E., & Bärtsch, P. (2007). Unchanged anaerobic and aerobic performance after short-term intermittent hypoxia. *Medicine and Science in Sports and Exercise, 39*(5), 858-864. doi:10.1249/mss.0b013e31803349d9

Teległów, A., Mardyła, M., Myszka, M., Pałka, T., Maciejczyk, M., Bujas, P., . . . Marchewka, J. (2022). Effect of Intermittent Hypoxic Training on Selected Biochemical Indicators, Blood Rheological Properties, and Metabolic Activity of Erythrocytes in Rowers. *Biology, 11*(10). doi:10.3390/biology11101513

Truijens, M., Rodríguez, F., Townsend, N., Stray-Gundersen, J., Gore, C., & Levine, B. (2008). The effect of intermittent hypobaric hypoxic exposure and sea level training on submaximal economy in well-trained swimmers and runners. *Journal of applied physiology (Bethesda, Md. : 1985), 104*(2), 328-337. doi:10.1152/japplphysiol.01324.2006

Truijens, M., Toussaint, H., Dow, J., & Levine, B. (2003). Effect of high-intensity hypoxic training on sea-level swimming performances. *Journal of applied physiology (Bethesda, Md. : 1985), 94*(2), 733-743. doi:10.1152/japplphysiol.00079.2002

Wonnabussapawich, P., Hamlin, M., Lizamore, C., Manimmanakorn, N., Leelayuwat, N., Tunkamnerdthai, O., . . . Manimmanakorn, A. (2017). Living and Training at 825 m for 8 Weeks Supplemented With Intermittent Hypoxic Training at 3,000 m Improves Blood Parameters and Running Performance. *Journal of Strength and Conditioning Research, 31*(12), 3287-3294. doi:10.1519/jsc.0000000000002227
